#  Loci cg06256735 and cg15815843 in the *MFAP5* gene regulatory regions are hypomethylated in varicose veins apparently due to active demethylation

**DOI:** 10.1042/BSR20231938

**Published:** 2024-05-29

**Authors:** Mariya A. Smetanina, Valeria A. Korolenya, Fedor A. Sipin, Igor P. Oscorbin, Kseniya S. Sevostyanova, Konstantin A. Gavrilov, Andrey I. Shevela, Maxim L. Filipenko

**Affiliations:** 1 Laboratory of Pharmacogenomics, Institute of Chemical Biology and Fundamental Medicine (ICBFM), Siberian Branch of the Russian Academy of Sciences (SB RAS), Novosibirsk 630090, Russia; 2 Department of Fundamental Medicine, V. Zelman Institute for Medicine and Psychology, Novosibirsk State University (NSU), Novosibirsk 630090, Russia; 3 Department of Natural Sciences, Novosibirsk State University (NSU), Novosibirsk 630090, Russia; 4 Center of New Medical Technologies, Institute of Chemical Biology and Fundamental Medicine (ICBFM), Siberian Branch of the Russian Academy of Sciences (SB RAS), Novosibirsk 630090, Russia; 5 Department of Surgical Diseases, V. Zelman Institute for Medicine and Psychology, Novosibirsk State University (NSU), Novosibirsk 630090, Russia

**Keywords:** DNA methylation, hydroxymethylcytosine, MFAP5 gene, varicose veins, vein layers

## Abstract

Varicose vein disease (VVD) is a common health problem worldwide. Microfibril-associated protein 5 (MFAP5) is one of the potential key players in its pathogenesis. Our previous microarray analysis revealed the cg06256735 and cg15815843 loci in the regulatory regions of the *MFAP5* gene as hypomethylated in varicose veins which correlated with its up-regulation. The aim of this work was to validate preliminary microarray data, estimate the level of 5-hydroxymethylcytosine (5hmC) at these loci, and determine the methylation status of one of them in different layers of the venous wall. For this, methyl- and hydroxymethyl-sensitive restriction techniques were used followed by real-time PCR and droplet digital PCR, correspondingly, as well as bisulfite pyrosequencing of +/- oxidized DNA. Our microarray data on hypomethylation at the cg06256735 and cg15815843 loci in whole varicose vein segments were confirmed and it was also demonstrated that the level of 5hmC at these loci is increased in VVD. Specifically, among other layers of the venous wall, *tunica (t.) intima* is the main contributor to hypomethylation at the cg06256735 locus in varicose veins. Thus, it was shown that hypomethylation at the cg06256735 and cg15815843 loci takes place in VVD, with evidence to suggest that it happens through their active demethylation leading to up-regulation of the *MFAP5* gene, and *t. intima* is most involved in this biochemical process.

## Introduction

Varicose vein disease (VVD) is the most common form of chronic vein disease that affects 33% of the population [1]. Risk factors for VVD are family history, age, gender, pregnancy, hormone replacement therapy, injury, occupation, exercise, constipation, obesity, diabetes, hypertension, smoking, and alcoholism [1,2]. Lack of attention to the VVD problem can lead to a number of complications that can threaten health and life, such as thrombophlebitis, external bleeding, and ulcers [3]. Oppression by symptoms increases the risk of depression compared with the general population [4]. In addition, advanced forms of the disease can lead to incapacity, disability, early retirement [5], and long-term health care costs [6]. For a more standardized approach to treatment, the CEAP classification of chronic vein diseases was compiled (Supplementary Table S1), taking into account Clinical, Etiological, Anatomical, and Pathophysiological features. The CEAP classification is regularly reviewed based on new clinical data [7].

**Figure 1:**
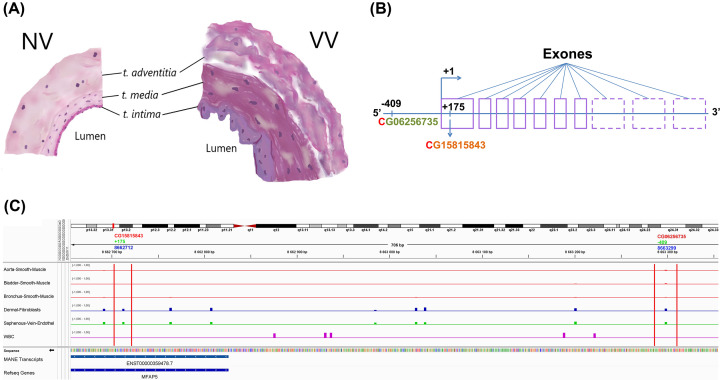
(A) Schematic representation of the cross-sections of normal (NV) and a varicose (VV) veins; (B) The location of the studied loci relative to the exons of the *MFAP5* gene; (C) *MFAP5* promoter methylation in various cell types **(B)** The broken line outlines exons that may not constitute an mRNA transcript; depending on the isoform, it may contain 7–10 exones. The cg15815843 locus is located in the first non-coding exon (in a proximal promoter region, +175 bases from the transcription start site); the cg06256735 locus is located in a distal promoter region (-409 bases from the transcription start site). **(C)** The figure was generated in the IGV browser. Cell types are indicated in the far-left panel, and methylation tracks are represented by different colors: red for smooth muscle cells, blue for fibroblasts, green for endotheliocytes (related to venous tissue), and violet for white blood cells (WBCs, as a distinct cell type not related to venous tissue). The two studied CpG dinucleotides are marked by red vertical lines. In the upper panel beneath the genome map, CpG numbers in the human genome are displayed in red color, coordinates from the *MFAP5* transcription start site are in green, and genome coordinates (hg38 assembly) are in blue. Note that transcription of the *MFAP5* gene occurs from right to left, and promoter coordinates are 12:8662789-8663388.

Different events occur in the venous wall during VVD. The vein wall consists of three functional layers with a certain cellular composition: *tunica* (*t*.) *intima*, *t. media* and *t. adventitia* (Figure 1A). *T. intima* is a single layer of endotheliocytes lying on the basement membrane and playing an important role in the perception of signals from the environment (blood), and responding to them [8]. *T. media* consists of three ordered layers of smooth muscle cells surrounded by collagen and elastin fibers, and performs a contractile and secretory function. *T. adventitia* is represented by collagen and, mainly, fibroblasts with a secretory phenotype. The extracellular matrix (ECM) maintains the integrity and homeostasis of the vein through interaction with cells of different layers of the vein wall [9]. During varicose transformation, hypertrophy of the intimal layer with subendothelial fibrosis is observed, as well as a change in the ECM composition and the phenotype of smooth muscle cells from contractile to secretory, the vein wall thickens [10].

It has been shown that epigenetic changes in the venous wall may be associated with the disease phenotype [11]. One of the mechanisms of transcription regulation is methylation/demethylation of cytosines (in the CpG site) in the regulatory regions of genes [12]. Methylation of the CpG site can enhance or prevent the binding of a transcription factor to its DNA motif, influencing (depending on the nature of a transcription factor) gene transcription [13], and it can also promote the formation of heterochromatin (thereby silencing gene expression) [14,15]. CpG methyl marks are symmetrically present on both DNA strands, so methylation patterns can be reproduced during replication [16]. DNA methylation and its maintenance is provided by the enzymes DNMT1 (DNA methyltransferase 1) [17] and UHRF1 (ubiquitin like with PHD and ring finger domains 1) [18]. Removal of methylated cytosines (5mC) can occur by passive and active mechanisms. Passive demethylation involves the loss of the methyl mark during replication without sufficient activity of the methylation maintenance system [19]. Active demethylation is catalyzed by enzymes of the TET (Ten-eleven translocation) family that stepwise oxidize 5mC to 5hmC (5-hydroxymethylcytosine), 5-formylcytosine and 5-carboxycytosin [20], which allows excision repair of the oxidized nucleotide with its replacement by cytosine [21].

In our previous work, the microarray analysis showed hypomethylation of the cg06256735 and cg15815843 loci (Figure 1B,C showing their genetic map) located in the regulatory region of the *MFAP5* gene (microfibril-associated protein 5) in varicose veins (VV), which correlated with increased expression of this gene [22]. Its product, MFAP5, is an ECM glycoprotein involved in microfibril assembly and elastogenesis [23], as well as in maintaining the integrity of large vessels [24]. In cholangiocarcinoma cells, an increased expression of the *MFAP5* is observed, which promotes the tube formation of human microvascular endothelial cells [25]. It has been shown that MFAP5 promotes angiogenesis and bone formation by activating the αvβ3/PTK2/AKT integrin signaling pathway [26]. The MFAP5 protein is able to bind the growth factors TGFβ and BMP, which may indicate its role in directed tissue-specific signaling that is performed by microfibrils in the ECM [24]. Overexpression of the *MFAP5* in cultured fibroblasts increases the stabilization of type I procollagen protein, which increases the type I collagen matrix [27] distinctive for VV [28]. Obesity-associated inflammation has been shown to increase *MFAP5* expression in adipose tissue [29].

In order to make reliable conclusions, any (epi)genome-wide analysis data need to be validated with an independent method using independent sample set. The aim of this work was to validate our previous findings on *MFAP5* promoter hypomethylation in VVD in an independent cohort and shed light on the potential mechanism. To this end, we aimed to profile DNA hydroxymethylation at these loci and assess the degree of methylation of one of them in different layers of the venous wall. For this, we used such methods as methyl-sensitive DNA restriction followed by real-time PCR, hydroxymethyl-sensitive restriction followed by droplet digital PCR, and pyrosequencing of bisulfite- and oxidative bisulfite-modified DNA (BS/oxBS-pyrosequencing). The essence of the first two methods is that the site-specific restriction endonuclease can or cannot cleave (depending on the restriction enzyme used) a DNA molecule with a modified cytosine, thus, the PCR amplification of the target DNA fragment is different in enzyme-treated and untreated samples. The third method involves the deamination of unmethylated cytosine to uracil. The readout of BS-pyrosequencing method is 5mC+5hmC. In parallel, oxBS-pyrosequencing is performed, where 5hmC is first chemically oxidized to 5-formylcytosine that is converted to uracil upon sodium bisulfite treatment [30]. The readout of oxBS-pyrosequencing method is therefore 5mC. To infer 5hmC (a marker of active demethylation), oxBS signal is subtracted from BS signal. Utilizing these precise independent methods for validation allows drawing firm conclusions to build a solid basis for future mechanistic studies.

## Methods

### Sample collection

Paired varicose vein (VV, case) and non-varicose vein (NV, control) segments (of the basin of the great saphenous vein) harvested from each patient with VVD were snap-frozen in liquid nitrogen during venous surgery. Exclusion criteria were post-thrombotic changes in deep veins on the leg with VV and absence of visible VV. Supplementary Tables S2–S7 show characteristics and numbers of patients in the samples.

### Separation of the venous wall into layers

Frozen vein segments were fixed in Tissue-Tek O.C.T. Compound (Sakura Finetek U.S.A.) and cut into cross sections (50 µm) using the freezing microtome CryoStar NX70 (Thermo Fisher Scientific, U.K., 2020). The sections were placed on Polysine® Slides (Thermo Fisher Scientific, Germany) and were fixed on the slide with staining (sequentially): 70% EtOH – 2 min; H_2_O – until O.C.T. is completely washed off (a few dips); cresyl violet acetate solution (0.5% cresyl violet acetate, 50% EtOH) – 30 s; excess stain was removed with absorbent pad; 70% EtOH – 10 s; 96% EtOH – 10 s; air-dry – until completely dry (2–3 min); freezing at −70°С (storage).

The resulting cross-section rings were dissected with needles under magnification into the venous layers. For DNA isolation, layer fragments were placed in a proteinase buffer (see ‘DNA isolation’ below).

### DNA isolation

Frozen vein segments or fragments of vein layers were placed in proteinase K buffer solution (1% sodium dodecyl sulfate, 1 mM CaCl_2_, 0.1 M NaCl, 2 mM EDTA, 50 mM Tris-HCl [рН 8.0], 0.25 mg/ml proteinase K [AppliChem GmbH, Germany]). The mixture was incubated overnight at 55°C. After proteinase K digestion followed by phenol/chloroform extraction and ethanol precipitation, samples were stored at −20°С [31]. DNA concentration was measured with a fluorimeter Qubit 4 using Qubit dsDNA HS (High Sensitivity) and Qubit dsDNA BR (Broad Range) Assay Kits.

### Esp3I restriction and Real-time quantitative PCR of restriction products

For the cg06256735 locus, a specific methyl-sensitive restriction endonuclease Esp3I was found using the Vector NTI 10.0.1 software (Invitrogen Corporation, U.S.A.). Esp3I restriction was carried out according to the manufacturer’s instructions (cat. No. ER0452, Thermo Fisher Scientific, U.S.A.). DNA from each sample was treated (‘Esp3I+’) / not treated (‘Esp3I-’) with a restriction enzyme that hydrolyzes only unmethylated recognition sites. This was followed by measuring the relative quantity of DNA with methyl tags in the studied locus in treated samples (‘Esp3I+’) and the total relative quantity of DNA (‘Esp3I-’) using real-time PCR.

Primers flanking the locus studied are shown in Supplementary Table S8. Reaction mixture: 64 mM Tris-HCl (pH 8.9), 24 mM (NH_4_)_2_SO_4_, 3 mM MgCl_2_, 0.025% Tween-20, 0.2 mM dNTP, 2 U Taq-pol (DNA-Synthesis, Russia), 0.3 µM primer mix, DNA sample/standard/control of equal concentration between the samples, SYBR Green-I was used as an intercalating dye. All reactions were carried out in triplicates. Amplification program (CFX-96 Thermal Cycler, Bio-Rad, U.S.A.): 3 min at 96°С, 40×: 10 s at 96°С, 10 s at 55°С, 10 s at 72°С, 10 s at 74°С to collect fluorescent signals at SYBR channel.

### Bisulfite conversion, PCR and pyrosequencing

The bisulfite conversion reaction was carried out in accordance with the recommendations of the manufacturer of the EZ DNA Methylation Kit (Zymo Research, U.S.A.). The mixture was purified using Zymo-Spin IC Columns at 12,000×***g*** for 30 s.

To amplify target DNA regions spanning over the cg06256735 and cg15815843 loci, PCR was performed before pyrosequencing. Primer sequences are shown in Supplementary Table S8. Reaction mixture consisted of 64 mM Tris-HCl (pH 8.9), 24 mM (NH_4_)_2_SO_4_, 3 mM MgCl_2_, 0.025% Tween-20, 0.2 mM dNTP, 2 U Taq-pol (DNA-Synthesis), 0.3 µM primer mix, DNA sample/standard/control of equal concentration between the samples. Amplification program (CFX-96 Thermal Cycler, Bio-Rad, U.S.A.): 15 min at 96°С, 50×: 10 s at 96°С, 30 s at 50°С, 30 s at 72°С. The results of PCR were evaluated using gel electrophoresis of the products in 8% polyacrylamide gel (in order to check their presence and size).

The pyrosequencing of PCR products was carried out using the PyroGold reagent kit (Biotage, U.S.A.) and the pyrosequencer PyroMark ID (Biotage, U.S.A.) according to the manufacturer’s instructions.

### AbaSI restriction and droplet digital PCR of restriction products

For the cg15815843 locus, a specific hydroxymethyl-sensitive restriction endonuclease AbaSI was found (New England Biolabs, U.S.A.). This enzyme selectively recognizes (on one or both strands) 5-glucosylhydroxymethylcytosine (5ghmC), 5-hydroxymethylcytosine (5hmC) but to a lower efficiency and 5mC to a negligible efficiency, introducing double-stranded DNA break, and does not recognize DNA with unmodified cytosine. To glycosylate 5hmC sites in the genome before AbaSI restriction, DNA (200 ng) was treated with T4 Phage beta-glucosyltransferase (T4-BGT) (cat. No. M0357S, New England Biolabs, U.S.A.) in the presence of UDP-glucose at 37°C overnight. T4-BGT was degraded by Proteinase K treatment at 50°C for 30 min followed by heat inactivation at 95°C for 10 min. Half of each reaction mixture was then digested with AbaSI (cat. No. R0665S) in 1xCutSmart buffer, according to the manufacturer's instructions (New England Biolabs, U.S.A.), followed by 20 min at 65°C to inactivate the enzyme. Untreated (‘AbaSI-’) samples were processed as treated samples (‘AbaSI+’) but without the addition of AbaSI. Equal amounts of DNA from the above reactions were used as templates for droplet digital PCR (ddPCR). ddPCR was performed with the QX100 Droplet Digital PCR system (Bio-Rad, U.S.A.). The ddPCR reaction mixture consisted of 10 µl of 2× ddPCR master mix (Bio-Rad, U.S.A.), approximately 10,000 copies of tested DNA, 0.9 µM primers, and 0.25 µM probe (Supplementary Table S8) in a final volume of 20 µl. All reactions were performed in duplicates. The entire reaction mixtures were loaded into a disposable plastic cartridge together with 70 µl of droplet generation oil and placed into the droplet generator. After processing, the droplets generated from each sample were transferred to a 96-well PCR plate (Eppendorf, Germany). PCR amplification was carried out in a Veriti thermal cycler (Applied Biosystems, U.S.A.) using a thermal profile of beginning at 95°C for 10 min, followed by 45 cycles of 94°C for 30 s, and 57°C for 60 s, and ending at 98°C for 10 min with ramp rate 2°C/s. After PCR, the plates were loaded on the droplet reader; and acquired data were analyzed with QuantaSoft Analysis Pro software (Bio-Rad, U.S.A.).

### DNA oxidation

To determine 5hmC level, the samples were subjected to an oxidation reaction before the bisulfite conversion. The oxidation reaction was carried out based on the procedures described in the literature [32,33]. The composition of the oxidizing agent: 15 mM KRuO_4_ in 0.05 M NaOH. After the completion of the reaction, the mixture was purified using Micro Bio-Spin P-30 Gel Columns in Tris buffer (Bio-Rad, U.S.A.) according to the manufacturer’s recommendations. To do this, the entire volume of the reaction mixture was applied to the prepared columns, centrifuged at 1,000×***g*** for 4 min. After that, the samples were processed as described in the paragraph ‘Bisulfite conversion, PCR and Pyrosequencing’.

### Estimation of 5hmC-to-U conversion efficiency after bisulfite treatment of oxidized synthetic 5hmC-dsDNA

To gain a measure of KRuO4-mediated efficiency of 5hmC-to-U conversion, Illumina next-generation sequencing was carried out on the synthetic dsDNA (100-nucleotide oligomer) containing 5hmC (Supplementary Table S8) after oxidative bisulfite treatment. First, the synthetic 5hmC control dsDNA from EpiMark® 5-hmC and 5-mC Analysis Kit (cat. No. E3317S, New England Biolabs, U.S.A.) was subjected to oxidation (the only difference was purification using Sephadex G-50 Ultra-Micro SpinColumn™ (cat. No. 74-7202, Harvard Bioscience, U.S.A.) according to the manufacturer’s protocol) and bisulfite conversion, as described above. The libraries were prepared in two technical repeats according to the protocol described by Kechin et al. [34]. The primers complementary to bisulfite-converted synthetic 5hmC control DNA and containing the adapter sequences including i5 and i7 index sequences (Illumina, U.S.A.) are shown in Supplementary Table S8. PCR amplifications of bisulfite-converted DNA were performed using EpiMark® Hot Start *Taq* DNA Polymerase (cat. No. M0490S, New England Biolabs, U.S.A.) according to the recommendations of the manufacturer. The results of PCR were evaluated using gel electrophoresis of the products in 8% polyacrylamide gel (in order to check their presence and size). After libraries purification and quantification, sequencing was performed on the MiniSeq System (Illumina, U.S.A.) with MiniSeq 300 Mid Output Reagent Kit (2x150 cycles). The sequencing data were analyzed by counting the numbers of reads containing/not containing bisulfite-converted nucleotides in the analyzed positions (see Supplementary Figure S1).

### Statistical analysis

Statistical analysis was performed using Microsoft Excel, STATISTICA, and Past statistical software. All the graphs were generated using Seaborn (data visualization library). The results of the calculations are presented by the median values of methylation percentage at the locus (М), the median pairwise NV/VV ratios of methylation percentage at the locus, the median pairwise VV/NV ratios of 5hmC percentage at the locus, and the *P*-value (*P*); tests were considered significant at *P*<0.05. The schematic representation of the analysis strategy and brief summary statistics are shown in Supplementary Figure S2.

Sample data distribution was determined using the Shapiro–Wilk, Kolmogorov–Smirnov, and Lilliefors normality tests. Summary statistics for all sample sets analyzed is presented in Supplementary Table S9. To visualize the distribution of values, box plots were constructed: the box borders show the interquartile range (values between the 1st and 3rd quartiles), the horizontal line inside it indicates the median, and the whiskers show the maximum and minimum values.

### Analysis of real-time PCR data after methyl-sensitive restriction and ddPCR data after hydroxymethyl-sensitive restriction

To detect differences in methylation of the *MFAP5*-associated cg06256735 locus between NV and VV using methyl-sensitive restriction enzyme, the ‘Esp3I+’ value was divided by the ‘Esp3I-’ value (paired values measured in parallel for each DNA sample), multiplied by 100, and the percentages of methylated cg06256735 locus obtained in whole vein segments (paired NV and VV for each patient) were compared with each other (NV vs. VV) using paired Wilcoxon test. Then the sample of whole vein segments was divided into two groups according to the clinical class [35]: ‘C2’ and ‘C3,4’ (C3 patients + C4 patients). Further the percentage values in whole vein segments (NV-VV pairs related to patients of different groups) were compared with each other using paired Student’s *t*-test or Wilcoxon test (depending on the normality).

The samples of vein layers were also compared in pairs separately for each layer (NV_I vs. VV_I, NV_M vs. VV_M, NV_A vs. VV_A, paired Student’s *t*-test). To confirm the differences in methylation at the studied locus in different layers of the vein wall in a certain condition, ANOVA (NV_I vs. NV_M vs. NV_A and VV_I vs. VV_M vs. VV_A) and Student’s *t*-test (unpaired comparisons) (VV_I vs. VV_M, VV_I vs. VV_A, VV_M vs. VV_A) were used.

AbasI-ddPCR data were analyzed as follows. The relative quantities of DNA after +/-AbaSI digestion were measured using droplet digital PCR (ddPCR) method. The proportional amount of 5hmC for each sample was calculated as a ratio of paired values measured in parallel for each DNA sample: the ‘AbasI-’ value (corresponding to the amount of input DNA with 5hmC tags, ddPCR processed data) was divided by the ‘AbasI+’ value (corresponding to the amount of DNA without 5hmC tags, ddPCR processed data). To detect differences in hydroxymethylation of the *MFAP5*-associated cg15815843 locus between NV and VV, the ratios obtained were compared with each other (paired NV vs. VV for each patient) using paired Wilcoxon test. Then the patient samples were divided into several groups according to the clinical class, age, gender, VVD manifestation, and BMI. Further, the relative 5hmC values in whole vein segments (NV-VV pairs related to patients of different groups) were compared with each other using paired Student’s *t*-test or Wilcoxon test (depending on the normality).

### Analysis of pyrosequencing data after bisulfite conversion (+/-DNA oxidation)

The pyrosequencing results were analyzed using the Pyro Q-CpG Software (version 1.0.9) (Biotage, U.S.A.). The principle of calculations to obtain the values of the percentage of methylated cytosines at the cg06256735 and cg15815843 loci in whole vein segments is presented in the methodological article [36]. The values obtained were compared (NV vs. VV) using the paired Student’s *t*-test and Wilcoxon test (depending on the normality).

In order to determine the percentage of 5hmC in the pool of 5mC + 5hmC at the cg06256735 and cg15815843 loci for each sample, the calculations were performed using the following formula:

$$[5\text{hmC}]=1-\left[\frac{\text{oxBS}}{\text{BS}}\right],$$

where ‘oxBS’ is the percentage of unconverted cytosines at the studied loci after oxidation and bisulfite conversion (only 5mC count), ‘BS’ is the percentage of unconverted cytosines at the studied loci after bisulfite conversion only (5mC and 5hmC count). The values obtained were compared (NV vs. VV) using the paired Student’s *t*-test.

### Multiple linear regression analysis

Multiple linear regression (MLR) analysis was performed using Microsoft Excel, STATISTICA, and Past statistical software. For correct variables combination and procedures we followed the recommendations described by Schneider et al. [37]. To avoid excessive collinearity and confounding, we excluded ‘Weight’ from the independent variable list and left ‘BMI’ instead (more appropriate one since it reflects weight adjusted to height, and also is not collinear to it). ‘Forward’ method was selected for a stepwise (or ridge) regression procedure. All the graphs were generated using STATISTICA and presented in Supplementary Figures S3–S8.

When performing MLR analysis vs. all parameters (independent predictor variables), all of them (Age, BMI, Gender, VVD manifestation, CEAP class, Height, VVD duration) available to us from the questionnaires with characteristics of patients participated in the study, were applied together in order to observe their possible effect on each of the dependent variables: ‘5mC/5hmC level in NV’, ‘5mC/5hmC level in VV’, or ‘5mC/5hmC NV:VV or VV:NV ratio’. Brief summary tables were incorporated in the corresponding Figures within the Results section. In case when some of the questionnaires with patient characteristics missed a few data (such as height and weight) we had to exclude those samples from MLR analysis (that is why there is an inconsistency between box plot graphs and MLR tables for ESP3I).

When performing MLR analysis vs. each particular predictor variable separately (such as Age or other one from the list) we used all three variables (‘5mC/5hmC level in NV’, ‘5mC/5hmC level in VV’, and ‘5mC/5hmC NV:VV or VV:NV ratio’) in order to visualize the results in one graph since there was no confounding between them.

The results of the calculations within all MLR analyses including all the models used are presented in Supplementary Table S10.

## Results

First, the data of the previous analysis [22] on hypomethylation at the cg06256735 (-409 from the transcription start site of the *MFAP5* gene) and cg15815843 (+175 from the transcription start site of the *MFAP5* gene) loci (Figure 1B,C) in VV were validated in whole vein segments by the independent methods (using methyl-sensitive restriction endonuclease and bisulfite conversion followed by pyrosequencing); the percentage of 5hmC at the cg06256735 and cg15815843 loci in NV and VV was also estimated. Then DNA methylation analysis of the cg06256735 locus in different layers of the venous wall was carried out. For comparing DNA methylation levels of the *MFAP5* promoter region (which contains both, cg06256735 and cg15815843, investigated loci) in different cell types related and not related to venous tissue, we utilized data from whole-genome bisulfite sequencing as presented in the study by Loyfer et al. [38] that focuses on the genome-wide DNA methylation atlas of human cells. We downloaded ‘*.bigwig’ files from the GEO dataset GSM5652176 for several cell types and aligned them in the IGV browser (Figure 1C).

### Determination of the methylation level at the cg06256735 and cg15815843 loci in whole vein segments

**Figure 2:**
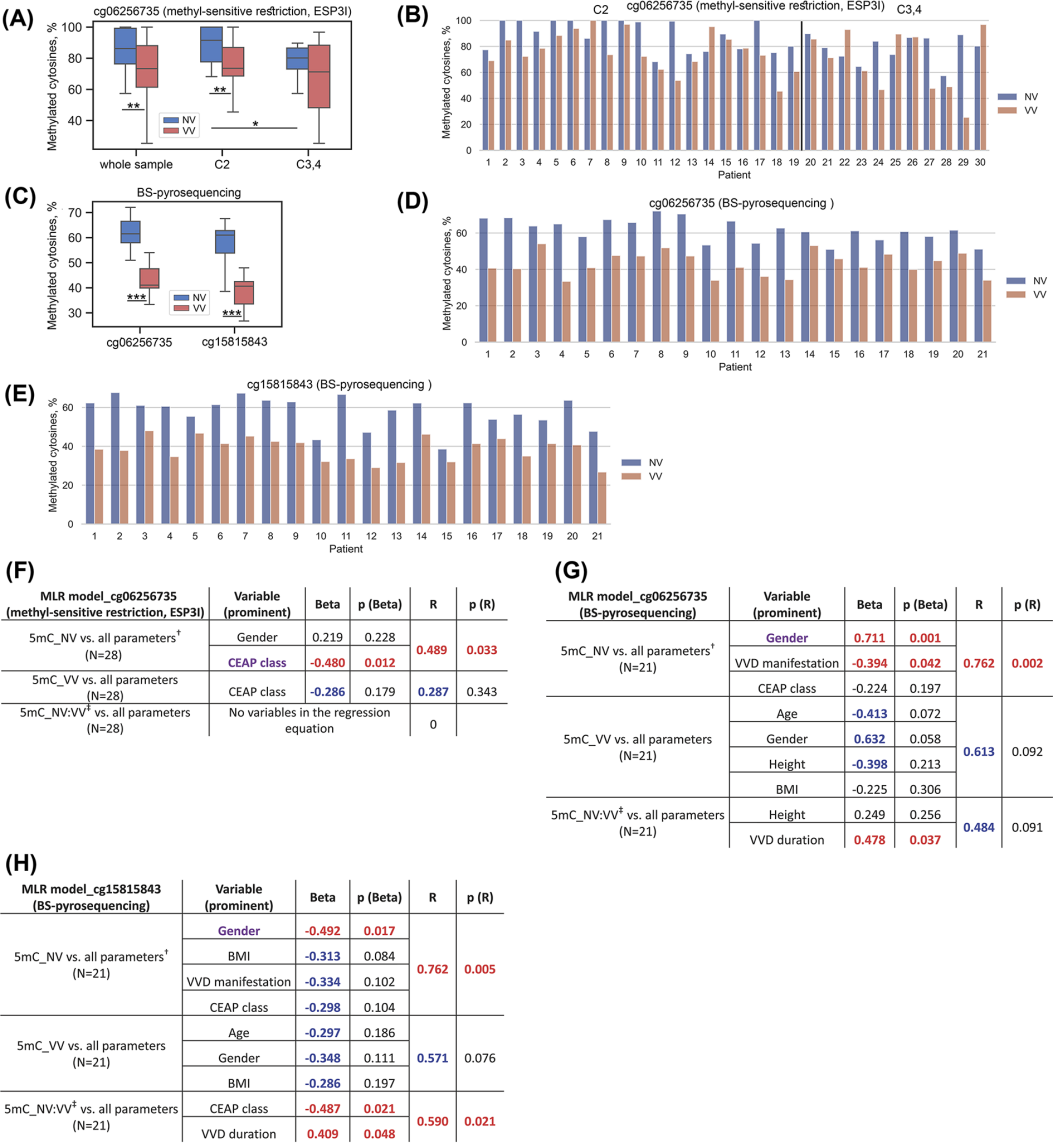
Analysis of data on the methylation status of the cg06256735 and cg15815843 loci (**A**) Distribution of percentage values of methylated cytosines at the cg06256735 locus in non-varicose (NV) vs. varicose vein (VV) segments obtained using the method of methyl-sensitive restriction (Esp3I); (**B**) Percentage values of methylated cytosines at the cg06256735 locus for each patient obtained using the method of methyl-sensitive restriction (Esp3I); (**C**) Distribution of percentage values of methylated cytosines at the cg06256735 and cg15815843 loci in NV vs. VV segments obtained by BS-pyrosequencing; (**D**) Percentage values of methylated cytosines at the cg06256735 locus for each patient obtained by BS-pyrosequencing; (**E**) Percentage values of methylated cytosines at the cg15815843 locus for each patient obtained by BS-pyrosequencing; (**F**) The result of multiple linear regression (MLR) analysis for the cg06256735 locus methylation data obtained using the method of methyl-sensitive restriction (Esp3I); (**G**) The result of multiple linear regression (MLR) analysis for the cg06256735 locus methylation data obtained by BS-pyrosequencing; (**H**) The result of multiple linear regression analysis (MLR) for the cg15815843 locus methylation data obtained by BS-pyrosequencing. 5mC, 5-methylcytosine; BS-pyrosequencing, bisulfite pyrosequencing; BMI, body mass index; C2/C3,4, clinical status according to CEAP classification; ESP3I, restriction enzyme; MLR, multiple linear regression; *N*, sample size; NV, non-varicose vein; VV, varicose vein; *P-value < 0.05, **P-value < 0.01, ***P-value < 0.001 (Wilcoxon test, paired Student’s *t*-test, Mann–Whitney *U* test); ^†^ All parameters (independent predictor variables) applied together: Age, BMI, Gender, VVD manifestation, CEAP class, Height, VVD duration; ^‡^ NV:VV ratio; predictor variables that had a significant effect on the corresponding dependent variable are displayed in purple color; Beta and R > |± 0.25| are displayed in blue color at *P*-value > 0.05; Beta and R > |± 0.25| are displayed in red color at *P*-value < 0.05; *P*-values < 0.05 are displayed in red color.

To validate the results of microarray analysis, we first analyzed the level of methylation of the cg06256735 locus using a methyl-sensitive restriction endonuclease (Esp3I) followed by PCR. As can be seen in Figure 2A, the percentage of methylated cg06256735 locus in NV is higher than in VV (1.09-fold: NV/VV = 1.09, NV(M: 86.57%) vs. VV(M: 77.14%), *P*=0.003). The distribution of values in paired NV and VV within patients could be also visualized in Figure 2B.

These results confirm, by an independent method, our previous microarray data on the change in the methylation status of the cg06256735 locus (hypomethylated in VV), which was also consistent with the change in the expression level of the *MFAP5* mRNA (up-regulated in VV) [22].

Pairwise comparison of NV with VV within the group of patients with a clinical class ‘C2’ (varicose veins) showed the expected ratio (NV/VV = 1.13, NV(M:98.65%) vs. VV(M:78.19%), *P*=0.001) (Figure 2A,B). Comparison within the group ‘C3,4’ (edema, skin changes) showed no significant difference (NV/VV = 1.05, NV (M:80.19%) vs. VV (M:71.26%), *P*=0.246). It is interesting to notice that in case of comparing the percentage values of methylated cytosines between only NV samples of two groups (‘C2’ vs. ‘C3,4’) the difference was observed (1.23-fold: NV(‘C2’)/NV(‘C3,4’) = 1.23, NV(‘C2’) (M:98.65%) vs. NV(‘C3,4’) (M:80.19%), *P*=0.022). Thus, difference in the methylation at the cg06256735 locus can be observed in different clinical statuses according to CEAP classification.

Since no commercially available methylation-sensitive restriction enzyme was found for the cg15815843 locus, an alternative method, pyrosequencing, was used. Additional validation of the cg06256735 locus methylation level was performed with pyrosequencing as well, which is more accurate compared with using restriction enzymes. As can be seen in Figure 2C-E, the percentages of methylated cg06256735 and cg15815843 loci in NV were significantly different from those in VV.

Both loci were hypomethylated in VV: 1.49-fold at cg06256735 (NV/VV = 1.49, NV(M:61.54%) vs. VV(M:41.08%), *P* = 9.54 × 10^−7^); 1.5-fold at cg15815843 (NV/VV = 1.5, NV(M:61.01%) vs. VV(M:40.65%), *P*=6 × 10^−5^). Interestingly, the methylation pattern observed at the cg06256735 locus was similar to that at the cg15815843 locus, which may point to their synergistic effect on the *MFAP5* gene expression.

This pyrosequencing analysis of the independent sample set confirmed the microarray analysis data on hypomethylation of the cg15815843 locus in VV, and additionally confirmed that for the cg06256735 locus.

To identify and characterize relationships among multiple factors (the level of 5mC in different vein types (NV or VV) or its ratio (NV:VV, since we observed hypomethylation in VV for both loci) and other parameters [independent predictor variables]), multiple linear regression (MLR) analysis was performed (see the Methods section for the details). Overall, MLR theoretically could enable us (a) to describe relationships among the dependent variables (such as 5mC level) and the independent variables (such as CEAP class, gender, age, etc.), (b) to estimate the values of the dependent variables from the observed values of the independent variables, and (c) to identify risk factors influencing the outcome and determine individual prognoses [37]. Full data table on all kinds of MLR analyses for all the models used is presented in Supplementary Table S10, and the graphs with correlations are presented in Supplementary Figures S3-S8.

According to the data obtained by the method of methyl-sensitive restriction (ESP3I) for the cg06256735 locus, CEAP class (displayed in purple, which means that addition of other variables in MLR did not change its single effect) had a reverse effect on the 5mC level in NV (Beta = −0.48, *P*(Beta) = 0.012; *R* = 0.489, *P*(*R*) = 0.033) (Figure 2F). It is consistent with the fact that 5mC level decreases in line with advanced (severe) stage of the disease. Other method (BS-pyrosequencing) showed the same trend of CEAP class for this locus in NV (Figure 2G) but not significant, though gender (Beta = 0.711, *P*(Beta) = 0.001) and VVD manifestation (Beta = −0.394, *P*(Beta) = 0.042) seemed to influence 5mC level in NV (*R* = 0.762, *P*(*R*) = 0.002). VVD duration tended to have a direct effect on the NV:VV ratio (Beta = 0.478, *P*(Beta) = 0.037; *R* = 0.484, *P*(*R*) = 0.091) pointing to more prominent hypomethylation of the cg06256735 locus in VV along with prolongation of the disease.

According to the data obtained by the method of BS-pyrosequencing for the cg15815843 locus, gender had a reverse effect on the 5mC level in NV (Beta = −0.492, *P*(Beta) = 0.017; *R* = 0.762, *P*(*R*) = 0.005) (Figure 2H). CEAP class and VVD duration had a reverse (Beta = −0.487, *P*(Beta) = 0.021) and direct (Beta = 0.409, *P*(Beta) = 0.048) effect, correspondingly, on the NV:VV ratio (*R* = 0.590, *P*(*R*) = 0.021) which is quite consistent with the data for another (cg06256735) locus.

### Determination of 5hmC level at the cg15815843 locus in whole vein segments using AbaSI-ddPCR

To estimate the level of 5hmC modification at the cg15815843 locus, hydroxymethyl-sensitive restriction enzyme AbaSI was used followed by quantification with droplet digital PCR - ddPCR (for details, see ‘AbaSI restriction and droplet digital PCR of restriction products’ in the Methods section). For another locus (cg06256735) such a commercially available enzyme was not found. It is known that although the levels of total 5hmC detected across the genome substantially vary across tissue types, these levels are approximately 14-fold lower than those of 5mC [39]. That is why we applied a ddPCR approach (as more sensitive and precise, compared to qPCR) that allows to detect small fold differences in target DNA copy number between the samples.

**Figure 3:**
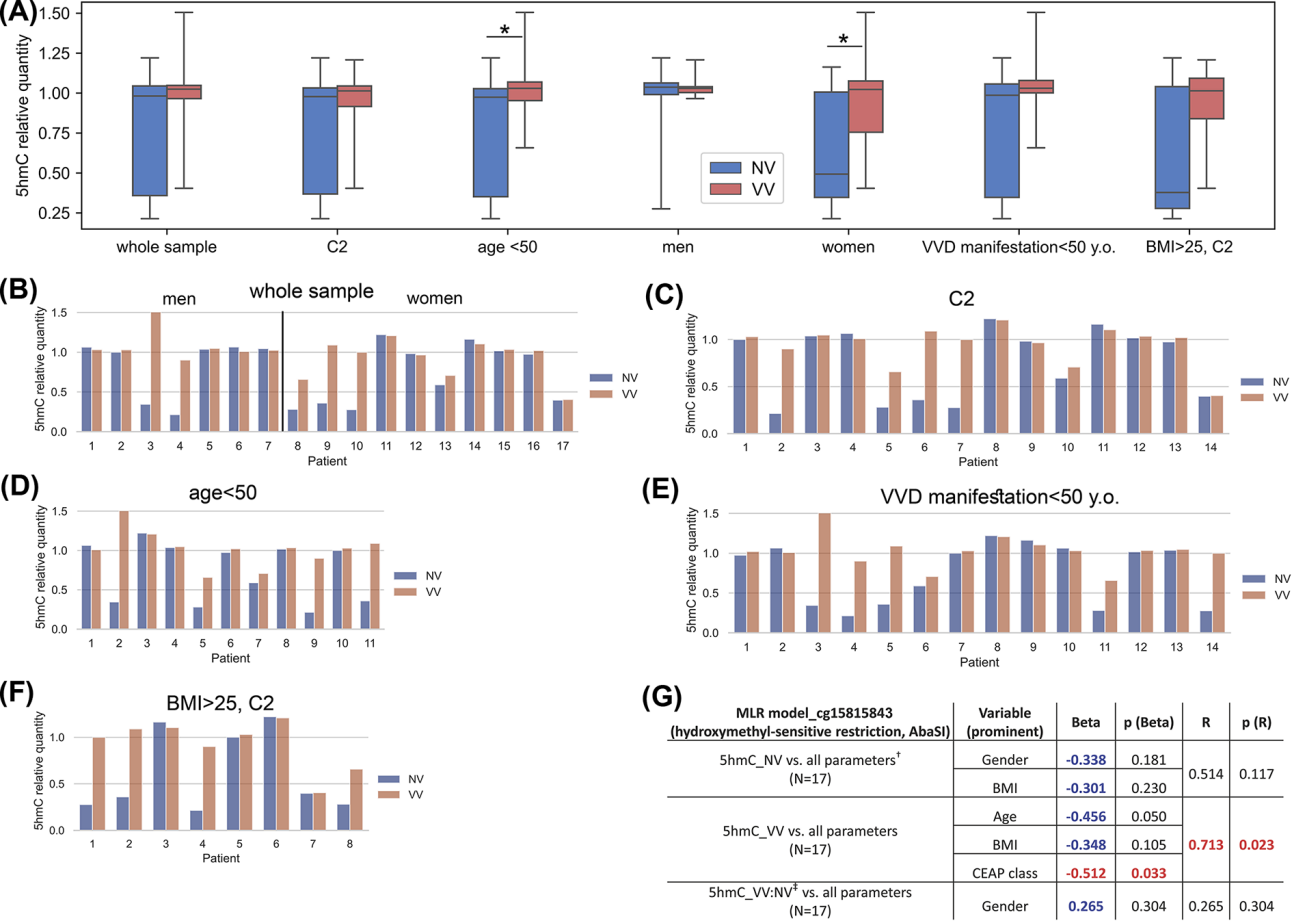
Analysis of data on the hydroxymethylation status of the cg15815843 locus obtained using the method of hydroxymethyl-sensitive restriction (AbaSI) (**A**) Distribution of proportional values of 5hmC in non-varicose (NV) vs. varicose (VV) vein segments in different subgroups of patients; (**B**) Proportional values of 5hmC for each patient (whole sample and gender subgroups); (**C**) Proportional values of 5hmC for each C2 patient; (**D**) Proportional values of 5hmC for each age<50 patient; (**E**) Proportional values of 5hmC for each VVD manifestation < 50 y.o. patient; (**F**) Proportional values of 5hmC for each BMI > 25, C2 patient; (**G**) The result of multiple linear regression (MLR) analysis for the cg15815843 locus hydroxymethylation data obtained using the method of hydroxymethyl-sensitive restriction (AbaSI); 5hmC, 5-hydroxymethylcytosine; AbaSI, restriction enzyme; BMI, body mass index; C2, clinical status according to CEAP classification; MLR, multiple linear regression; NV, non-varicose vein; VV, varicose vein; *N*, sample size; *P-value < 0.05 (Wilcoxon test, paired Student’s *t*-test); ^†^ all parameters (independent predictor variables) applied together: Age, BMI, Gender, VVD manifestation, CEAP class, Height, VVD duration; ^‡^ VV:NV ratio; Beta and *R* > |± 0.25| are displayed in blue color at *P*-value > 0.05; Beta and *R* > |± 0.25| are displayed in red color at *P*-value < 0.05; *P*-values < 0.05 are displayed in red color.

To detect differences in hydroxymethylation of the *MFAP5*-associated cg15815843 locus between NV and VV, the ratios obtained were compared with each other (paired NV vs. VV for each patient) using paired Wilcoxon test. As one can see in Figure 3A,B, statistical significance was not reached to show any difference in 5hmC level at the cg15815843 locus between the samples within the whole sample set (*P*=0.102), although within ‘C2’ patient group (Figure 3A,C) we observed a tendency (not significant though, *P*=0.074).

Then the sample of whole vein segments was divided into several groups according to the age, gender, VVD manifestation, and BMI. Comparison within the group of patients aged less than 50 years (Figure 3A,D) showed a slight but significant increase in the level of 5hmC at the cg15815843 locus in VV (VV/NV = 1.05, NV (M:0.97) vs. VV (M:1.03), *P*=0.026). After stratification the sample by gender, statistically significantly increased 5hmC level was found only in VV of women (1.12-fold: VV/NV = 1.12, NV (M:0.49) vs. VV (M:1.02), *P*=0.035) (Figure 3A,B) but not of men (*P*=0.81), suggesting that *MFAP5* is related to the involvement of sex hormones in VVD pathogenesis [40]. When comparing paired NV vs. VV samples within the group of VVD manifestation under 50 years old (Figure 3A,E), we revealed a tendency for an increase in the level of 5hmC in VV (VV/NV = 1.04, NV (M:0.99) vs. VV (M:1.03), *P*=0.056), suggesting that changes in the degree of hydroxymethylation at the cg15815843 locus do not aggravate with age. Comparison within ‘C2’ patient group with BMI>25 (Figure 3A,F) also showed a tendency for an increase in the level of 5hmC in VV (VV/NV = 1.69, NV (M:0.38) vs. VV (M:1.01), *P*=0.093), confirming BMI as a risk factor for VVD development [1].

Further we applied MLR analysis to the data obtained by the method of hydroxymethyl-sensitive restriction (AbaSI) for the cg15815843 locus. CEAP class had a reverse effect on the 5hmC level in VV (Beta = −0.512, *P*(Beta) = 0.033; *R* = 0.713, *P*(*R*) = 0.023) (Figure 3G).

### Determination of 5hmC level at the cg06256735 and cg15815843 loci in whole vein segments using BS/oxBS-pyrosequencing

The pyrosequencing method we chose also allowed us to distinguish 5hmC from 5mC due to additional treatment procedure (see ‘DNA oxidation’ of the Methods section). The efficiency of oxidation and bisulfite conversion was checked using Illumina NGS technology (see the Methods section). An overall 5hmC-to-U conversion level of 93.21% was observed (Supplementary Figure S1). The efficiency of bisulfite conversion of unmethylated cytosines nearby the 5hmC site (to the left and to the right, excluding primer annealing regions) varied between 99.5% and 99.88%, and the average efficiency was 99.72%. In the work of Booth et al. [33] where they presented the thoroughly tested and adapted 5hmC quantitative sequencing protocol, estimated bisulfite conversion rates varied between 99.8% and 99.9%, and the total level of 5hmC-to-U conversion was 94.5%, which is consistent with our data.

**Figure 4:**
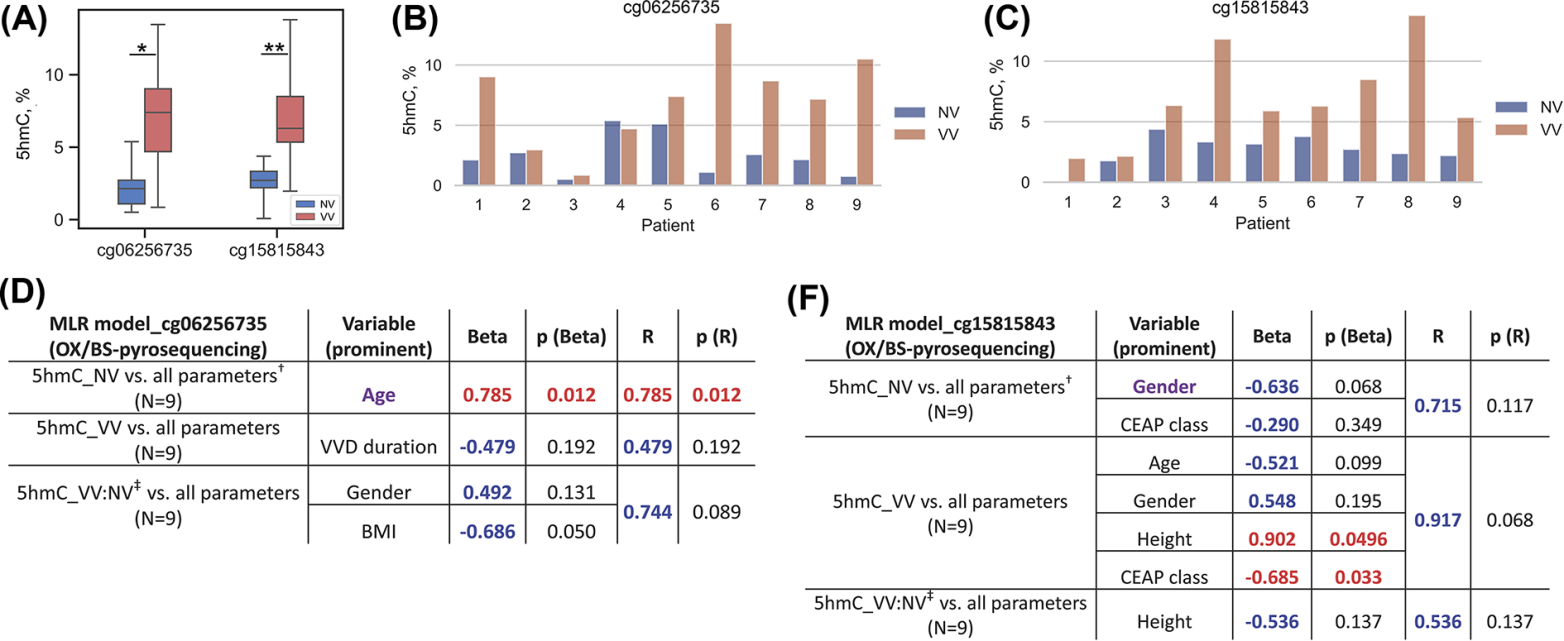
Analysis of data on the hydroxymethylation status of the cg06256735 and cg15815843 loci obtained by BS/oxBS-pyrosequencing (**A**) Distribution of percentage values of 5hmC at the cg06256735 and cg15815843 loci in non-varicose (NV) vs. varicose (VV) vein segments; (**B**) Percentage values of 5hmC at the cg06256735 locus for each patient. (**C**) Percentage values of 5hmC at the cg15815843 locus for each patient. (**D**) The result of multiple linear regression (MLR) analysis for the cg06256735 locus hydroxymethylation data. (**E**) The result of multiple linear regression (MLR) analysis for the cg15815843 locus hydroxymethylation data. NV, non-varicose vein; VV, varicose vein; CEAP, classification; OX/BS-pyrosequencing, bisulfite pyrosequencing with pre-oxidation; MLR, multiple linear regression; BMI, body mass index; 5hmC, 5-hydroxymethyl-cytosine; *N*, sample size; *P-value < 0.05, **P-value < 0.01 (paired Student’s *t*-test); ^†^ all parameters (independent predictor variables) applied together: Age, BMI, Gender, VVD manifestation, CEAP class, Height, VVD duration; ^‡^ VV:NV ratio; predictor variables that had a significant effect on the corresponding dependent variable are displayed in purple color; Beta and *R* > |± 0.25| are displayed in blue color at *P*-value > 0.05; Beta and *R* > |± 0.25| are displayed in red color at *P*-value < 0.05; *P*-values < 0.05 are displayed in red color.

The analysis of BS/oxBS-pyrosequencing data showed an increase in the percentage of 5hmC (in the pool of 5mC + 5hmC) at the cg06256735 and cg15815843 loci in VV (Figure 4A-C). Thus an increase in the level of 5hmC in VV was: 3.34-fold at the cg06256735 locus (VV/NV = 3.34, NV(M:2.14%) vs. VV(M:7.39%), *P*=0.018) and 2.43-fold at the cg15815843 locus (VV/NV = 2.43, NV(M:2.71%) vs. VV(M:6.30%), *P*=0.002). As with methylation, the levels of 5hmC at the cg06256735 locus in NV and VV were similar to those at the cg15815843 locus. An increased level of 5hmC at these loci in VV condition can point to their active DNA demethylation [41] in the venous wall DNA during varicose transformation.

We also applied MLR analysis to BS/oxBS-pyrosequencing data for both (cg06256735 and cg15815843) loci. Age (displayed in purple, which means that addition of other variables in MLR did not change its single effect) had a direct effect on the level of 5hmC at the cg06256735 locus in NV (Beta = 0.785, *P*(Beta) = 0.012; *R* = 0.785, *P*(*R*) = 0.012) (Figure 4D) meaning that elevated 5hmC level may correlate with vascular age (since 5hmC modification was accumulated in elderly veins). For the cg15815843 locus (Figure 4E) gender tended to have an effect on the 5hmC level in NV (*R* = 0.715, *P*(*R*) = 0.117), height and CEAP class – in VV (*R* = 0.917, *P*(*R*) = 0.068) but all of them did not reach statistical significance of R coefficient.

### Determination of the methylation level at the cg06256735 locus in different layers of the vein wall

Based on the fact that the venous wall is heterogeneous (consisting of different cell populations) [10], we studied, using methylation-sensitive restriction enzyme (Esp3I), the degree of methylation at the cg06256735 locus in different layers of the venous wall (*t. intima*, *t. media*, and *t. adventitia*, designated hereafter as “I, M, A”).

**Figure 5:**
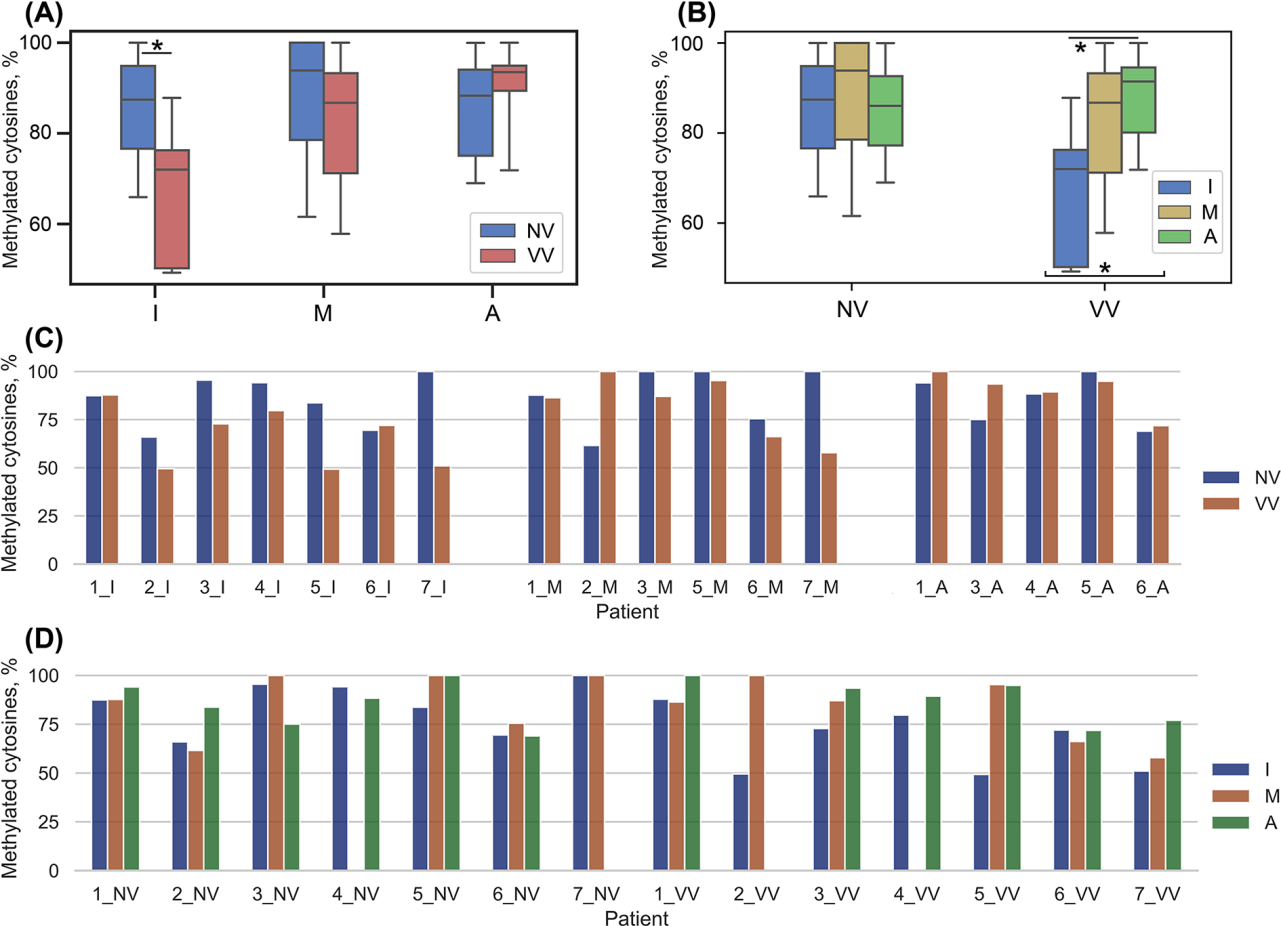
Analysis of data on the methylation status of the cg06256735 locus obtained using the method of methyl-sensitive restriction in the layers of the venous wall (**A**) Distribution of percentage values of methylated cytosines in each layer of non-varicose (NV) and varicose (VV) vein segments (paired sample, paired Student’s *t*-test); (**B**) Distribution of percentage values of methylated cytosines in each layer of NV and VV segments (all sample, ANOVA, Student’s *t*-test); (**C**) Percentage values of methylated cytosines at the cg06256735 locus in NV and VV segments for each patient and each layer (paired samples); (**D**) Percentage values of methylated cytosines at the cg06256735 locus in NV and VV segments for each patient and each layer (all samples). NV, non-varicose vein; VV, varicose vein; I - *t. intima*; M - *t. media*; A - *t. adventitia*.; *P-value < 0.05.

The analysis showed that when comparing *t. intima* of the NV vs. VV, there was 1.31-fold decrease in methylation at the cg06256735 locus in VV: NV_I/VV_I = 1.31, NV_I(M:87.43%) vs. VV_I(M:72.00%), *P*=0.033 (Figure 5A,C).

For the other layers, no significant difference was shown in the level of the cg06256735 locus methylation: NV_M/VV_M = 1.10, NV_M(M:93.86%) vs. VV_M(M:86.74%), *P*=0.531; NV_A/VV_A = 0.96, NV_A(M:88.32%) vs. VV_A(M:93.52%), p = 0.313. We do not exclude the fact that hypomethylation of the cg06256735 locus in *t. media* may contribute to the overall hypomethylation status of this locus throughout the venous wall.

Then the difference in methylation status at the cg06256735 locus between the layers of the vein wall in a particular condition (NV or VV) was investigated (Figure 5B,D). This analysis showed differences in the percentage of methylated cytosines at the locus in VV: VV_I(72.00%) vs. VV_M(86.74) vs. VV_A(91.45%), *P*=0.044 (ANOVA [analysis of variance]).

When comparing three layers of the NV wall, there was no difference in the percentage of methylation at the cg06256735 locus (NV_I(87.43%) vs. NV_M(93.86%) vs. NV_A(86.04%), *P*=0.941), while in case of VV there was a difference in methylation at this locus between the layers. In VV condition, the cg06256735 locus in *t. intima* was significantly hypomethylated compared to that in *t. adventitia* (VV_I(M:72.00%) vs. VV_A(M:91.45%), *P*=0.02). In NV condition, there was a tendency (not significant though) toward a lower level of methylation at the cg06256735 locus in *t. intima* compared to *t. media* (VV_I (M:72.00%) vs. VV_M(M:86.74%), *P*=0.097). At this point we could not perform MLR analysis because of the limited sample size.

It can be concluded that there is a shift in the methylation status at the studied locus in different layers of the venous wall during VVD. Thus, the microarray data on hypomethylation at the cg06256735 and cg15815843 loci in VVD were confirmed using independent methods and sample sets; differences in 5hmC level at both loci between NV and VV, as well as differences in methylation level at the cg06256735 locus in different layers of the vein wall, were shown.

## Discussion

Epigenetic changes in the venous wall play a role in the pathogenesis of VVD [11], in particular, methylation/demethylation of CpGs in the regulatory regions of genes [22]. DNA methylation plays a huge role: this process is involved not only in the regulation of gene expression, but also in the silencing of transposable elements, genomic imprinting, and inactivation of the X-chromosome. High levels of CpG site methylation (70–80% in somatic cells) are observed in the mammalian genome [42].

In our study, we confirmed demethylation at the cg06256735 and cg15815843 sites in the regulatory regions of the *MFAP5* gene in VVD using methyl-specific restriction and pyrosequencing methods. The product of this gene is involved in the processes related to VV [43]: inflammation [29], ECM remodeling [23,27], cell signaling disorder [24,26], hypoxia [44]. In patients with ‘C2’ clinical class (varicose veins), hypomethylation of the cg06256735 locus was shown; moreover, it was more prominent compared with such an effect observed within the whole sample set including both subgroups ‘C2’ and ‘C3,4’; in ‘C3,4’ patients (edema, skin changes) the tendency to its hypomethylation was not significant (*P*>0.1). This result indicates that in patients with a more severe class of disease (C3,4 compared with C2), in addition to the varicose vein, a conditionally healthy vein also becomes involved in the process of hypomethylation. All this allows us to assume that hypomethylation of the regulatory region of the MFAP5 gene may be a marker of this disease and can also be observed in conditionally healthy vessels during its development.

The active mechanism of demethylation involves the chemical modification of 5mC with the help of TET proteins, and the more quantitatively expressed catalysis product of these enzymes, 5hmC, can be considered a marker of this process [20]. Although 5mC may promote heterochromatin formation (which prompts the silencing of gene expression), it is suggested that 5hmC is mainly associated with euchromatin and gene activity [45]. At first instance, we attempted to quantify 5hmC modification at the cg15815843 using AbaSI-ddPCR. AbaSI restrictase has its limitations, thus using it could not guarantee a 100% reliable detection of 5hmC tags avoiding false-positive results. The activity of this enzyme is too complex [46] since it cleaves DNA containing 5mC to a negligible extent: 500-fold less efficiently than DNA containing 5hmC and 8000-fold less efficiently than DNA containing 5ghmC [47]. That could possibly level off 5hmC values between NV and VV samples, and ultimately did not allow us to reach desirable statistical significance using that particular method. Using another, in our opinion more precise method of BS/ox-BS-pyrosequencing, we showed an increase of the level of 5hmC (in the pool of 5mC + 5hmC) at the cg06256735 and cg15815843 loci, which may indicate the process of active demethylation of these loci in VVD, leading to an elevated transcription of the *MFAP5* gene in VV [22] confirmed by the previous study. Based on the pyrosequencing data that showed similar patterns of methylation and 5hmC levels at both loci, one can suggest a co-regulation of the *MFAP5* gene transcription by changing their methylation status. Another supportive argument to our findings is that the loci we investigated do not belong to CpG islands in which 5hmC is generally depleted [48]. Since it is widely accepted that multiple epigenetic modifications may have a synergistic effect on gene transcription alteration [49], these loci, in addition to other proximal or distal regulatory regions, may represent a target for regulatory machinery.

The results of multiple linear regression (MLR) analyses (for different models depending on the datasets used) enabled us to extract additional information regarding the association of various parameters (predictor variables) with the (hydroxy)methylation status of the studied loci in veins. As such, we observed a consistency of the 5mC level decrease at the cg06256735 locus in VVD with patient’s BMI and advanced (severe) stage of the disease. Also, accumulation of 5hmC modification at the cg06256735 locus was observed in elderly veins pointing to its possible correlation with vascular age. Gender influenced the 5mC level at both loci.

The layers of the venous wall fulfill certain functions, so it is logical to assume their different contributions to the processes of VVD development. Comparing each of 3 layers between the conditions (NV vs. VV), hypomethylation at the cg06256735 locus was shown only in *t. intima* of VV. In addition, comparison between the vein wall layers in a particular condition (VV or NV) showed a difference at the cg06256735 methylation status between them in VV. Specifically, *t. intima* differs markedly from *t. adventitia* and also tends to differ from *t. media* (*P*<0.1), which makes it standing out from other layers. It has been demonstrated that *t. intima* is the main contributor to hypomethylation at the cg06256735 locus in whole vein segments. The endotheliocytes lining up this layer play a central role in maintaining vascular homeostasis [50]. Some signs of pathologically changed *t. intima* are hypertrophy, inclusions of collagen, inflammation [43], which may be associated with increased expression of the *MFAP5* in VV [22]. As already mentioned, MFAP5 has the ability to bind growth factors [24], possibly regulating tissue growth; it also increases the stability of type I procollagen, which can contribute to the formation of collagen inclusions; it is related to inflammation [29]. In addition, there are data on the ability of the endothelium to respond to ECM stiffness [51], and MFAP5, in its turn, is involved in the assembly of microfibrils and elastogenesis [23]. Thus, an important role of hypomethylation of the cg06256735 locus in *t. intima* of VVD phenotype can be assumed.

Our work somehow is not without limitations. First, this study was based on the small sample sets of patients to investigate the level of 5hmC in the studied loci in whole vein segments and the level of methylation at the cg06256735 locus in the layers of the venous wall because of the limited amount of clinical postoperative material. Due to this reason, it was practically impossible to conduct different types of analyses on the same sample set for a deeper study of the processes. On the other hand, potential limitations for the hydroxymethyl-sensitive restriction experiments could easily stem from the enzymatic properties of AbaSI. Indeed, since the levels of 5hmC are low and vary in different cell types, the interpretation of 5hmC data can be significantly influenced by the sensitivity of methods and technologies used [39]. Moreover, as it is usually the case in human studies, there are many sources of inter-individual variability between subjects and groups [49] and the high level of inter-individual variability in 5hmC, in particular, consistently being reported, in our experimental data we also observed that phenomenon. Strictly speaking, in order to be generalized, our findings need to be replicated in different patient populations.

Thus, we validated our microarray analysis preliminary data on hypomethylation at the cg06256735 and cg15815843 loci located in the *MFAP5* gene regulatory regions in VVD associated with up-regulation of this gene. Using BS/oxBS-pyrosequencing we showed the increased level of 5hmC (in the pool of 5mC+5hmC) at these loci which serves a marker of active demethylation. Our data suggest that 5hmC is involved in pathological processes related to varicose vein condition. We also identified the layer of the venous wall - *t. intima* - the main contributor to hypomethylation at the cg06256735 locus in whole vein segments in VVD.

## Supplementary Material

Supplementary Figures S1-S8 and Tables S1-S10

## Data Availability

Data are contained within the article and Supplementary Materials, and available from the corresponding author on reasonable request.

## Abbreviations

5ghmC: 5-glucosylhydroxymethylcytosine
5hmC: 5-hydroxymethylcytosine
MFAP5: microfibril-associated protein 5
MLR: multiple linear regression
NV: non-varicose vein
VV: varicose vein
VVD: varicose vein disease
