# Supplementary material for: Loci cg06256735 and cg15815843 in the MFAP5 gene regulatory regions are hypomethylated in varicose veins apparently due to active demethylation
Source: Biosci Rep. 2024 May 29;44(5):BSR20231938. doi: 10.1042/BSR20231938 (PMC11139664; doi:10.1042/BSR20231938)
Supplement: Supplementary Figures S1-S8 and Tables S1-S10 [file BSR-2023-1938_supp.pdf]

Table S1. List of clinical statuses of patients with chronic venous disease according to the CEAP classification.

Clinical class	Description
C0	No visible or palpable signs of venous disease
C1	Telangiectasias or reticular veins
C2	Varicose veins
C3	Edema
C4	Changes in skin and subcutaneous tissue secondary to chronic venous disease
C5	Healed venous ulcers
C6	Active venous ulcers

Table S2. Characteristics of patients participated in the study to determine percentage values of methylated cytosines at the cg06256735 locus (method of methyl-sensitive restriction, ESP3I).

**sex**	**women**		**men**	
	21		9	
**clinical status**	**C2**	**C3**		**C4**
	17	9		4
**family history**	**positive**	**none**		**unknown**
	19	9		2
**comorbidities**	**obesity**	**hypothyroidism**	**cardiovascular diseases**	**none**
	2	1	5	20
	**median (range)**			
**age, years**	48.5 (27 – 69)			
**VVD onset, years**	30 (10 – 55)			
**VVD duration, years**	15 (3 – 39)			
**BMI, kg/m^2**	25.63 (17.99 – 42.86)			

Table S3. Characteristics of patients participated in the study to determine percentage values of methylated cytosines at the cg06256735 and cg15815843 loci (pyrosequencing).

**sex**	**women**		**men**	
	15		5	
**clinical status**	**C2**	**C3**		**C4**
	15	4		1
**family history**	**positive**	**none**		**unknown**
	14	3		3
**comorbidities**	**cardiovascular diseases**	**diabetes**	**peptic ulcer of the duodenum**	**none**
	6	3	3	9
	**median (range)**			
**age, years**	42 (28 - 70)			
**VVD onset, years**	22 (10 - 55)			
**VVD duration, years**	19 (1 - 40)			
**BMI, kg/m^2**	24.68 (19.39 – 42.02)			

Table S4. Characteristics of patients participated in the study to determine relative quantity of 5hmC at the cg15815843 locus (method of hydroxymethyl-sensitive restriction, AbaSI).

<table>
<tr><th rowspan="2">sex</th><th colspan="3">women</th><th colspan="3">men</th></tr>
<tr><td colspan="3">10</td><td colspan="3">7</td></tr>
<tr><th rowspan="2">clinical status</th><th colspan="2">C2</th><th colspan="2">C3</th><th colspan="2">C4</th></tr>
<tr><td colspan="2">14</td><td colspan="2">3</td><td colspan="2">0</td></tr>
<tr><th rowspan="2">family history</th><th colspan="2">Positive</th><th colspan="2">none</th><th colspan="2">unknown</th></tr>
<tr><td colspan="2">11</td><td colspan="2">2</td><td colspan="2">3</td></tr>
<tr><th rowspan="2">comorbidities</th><th>cardiovascular diseases</th><th>erythremia</th><th>gastrointestinal disease</th><th>metabolic diseases</th><th>obesity</th><th>none</th></tr>
<tr><td>4</td><td>1</td><td>4</td><td>2</td><td>2</td><td>5</td></tr>
<tr><td></td><th colspan="6">median (range)</th></tr>
<tr><th>age, years</th><td colspan="6">46 (30 – 70)</td></tr>
<tr><th>VVD onset, years</th><td colspan="6">27 (15 – 55)</td></tr>
<tr><th>VVD duration, years</th><td colspan="6">15 (1 – 40)</td></tr>
<tr><th>BMI, kg/m^2</th><td colspan="6">25.21 (19.39 – 42.02)</td></tr>
</table>

Table S5. Characteristics of patients participated in the study to determine percentage values of 5hmC at the cg06256735 locus (BS/oxBS-pyrosequencing).

<table>
<tr><th rowspan="2">sex</th><th colspan="2">women</th><th colspan="3">men</th></tr>
<tr><td colspan="2">6</td><td colspan="3">3</td></tr>
<tr><th rowspan="2">clinical status</th><th>C2</th><th colspan="2">C3</th><th colspan="2">C4</th></tr>
<tr><td>7</td><td colspan="2">2</td><td colspan="2">0</td></tr>
<tr><th rowspan="2">family history</th><th>positive</th><th colspan="2">none</th><th colspan="2">unknown</th></tr>
<tr><td>8</td><td colspan="2">1</td><td colspan="2">0</td></tr>
<tr><th rowspan="2">accompanying illnesses</th><th>cardiovascular diseases</th><th>erythremia</th><th colspan="2">peptic ulcer of the duodenum</th><th>none</th></tr>
<tr><td>3</td><td>1</td><td colspan="2">1</td><td>4</td></tr>
<tr><td></td><th colspan="5">median (range)</th></tr>
<tr><th>age, years</th><td colspan="5">37 (28 – 70)</td></tr>
<tr><th>VVD onset, years</th><td colspan="5">20 (10 – 36)</td></tr>
<tr><th>VVD duration, years</th><td colspan="5">18 (1 – 40)</td></tr>
<tr><th>BMI, kg/m^2</th><td colspan="5">22.60 (19.39 – 28.38)</td></tr>
</table>

Table S6. Characteristics of patients participated in the study to determine percentage values of 5hmC at the cg15815843 locus (BS/oxBS-pyrosequencing).

<table>
<tr><th rowspan="2">sex</th><th colspan="2">women</th><th colspan="3">men</th></tr>
<tr><td colspan="2">5</td><td colspan="3">4</td></tr>
<tr><th rowspan="2">clinical status</th><th>C2</th><th colspan="2">C3</th><th colspan="2">C4</th></tr>
<tr><td>7</td><td colspan="2">2</td><td colspan="2">0</td></tr>
<tr><th rowspan="2">family history</th><th>positive</th><th colspan="2">none</th><th colspan="2">unknown</th></tr>
<tr><td>8</td><td colspan="2">0</td><td colspan="2">1</td></tr>
<tr><th rowspan="2">comorbidities</th><th>cardiovascular diseases</th><th>erythremia</th><th colspan="2">peptic ulcer of the duodenum</th><th>none</th></tr>
<tr><td>3</td><td>1</td><td colspan="2">2</td><td>4</td></tr>
<tr><td></td><th colspan="5">median (range)</th></tr>
<tr><th>age, years</th><td colspan="5">37 (28 – 70)</td></tr>
<tr><th>VVD onset, years</th><td colspan="5">20 (10 – 55)</td></tr>
<tr><th>VVD duration, years</th><td colspan="5">18 (1 – 40)</td></tr>
<tr><th>BMI, kg/m^2</th><td colspan="5">22.60 (19.39 – 27.73)</td></tr>
</table>

Table S7. Characteristics of patients participated in the study to determine percentage values of methylated cytosines at the cg06256735 locus in different layers of the venous wall (method of methyl-sensitive restriction, ESP3I).

<table>
<tr><td rowspan="2">sex</td><td colspan="2">women</td><td colspan="2">men</td></tr>
<tr><td colspan="2">7</td><td colspan="2">0</td></tr>
<tr><td rowspan="2">clinical status</td><td>C2</td><td colspan="2">C3</td><td>C4</td></tr>
<tr><td>5</td><td colspan="2">2</td><td>0</td></tr>
<tr><td rowspan="2">family history</td><td>positive</td><td colspan="2">none</td><td>unknown</td></tr>
<tr><td>3</td><td colspan="2">1</td><td>3</td></tr>
<tr><td rowspan="2">comorbidities</td><td>obesity</td><td>hypothyroidism</td><td>arterial hypertension</td><td>none</td></tr>
<tr><td>1</td><td>1</td><td>1</td><td></td></tr>
<tr><td></td><td colspan="4">median (range)</td></tr>
<tr><td>age, years</td><td colspan="4">43 (26 –65)</td></tr>
<tr><td>VVD onset, years</td><td colspan="4">30 (17 – 50)</td></tr>
<tr><td>VVD duration, years</td><td colspan="4">9 (6 – 19)</td></tr>
<tr><td>BMI, kg/m^2</td><td colspan="4">23.26 (20.52 – 34.20)</td></tr>
</table>

Table S8. Primers used in the study.

5’- GAAGAGTCTAAATGTGTAGGTCAAG -3’<br>5’- TTCCATATTTGAATCATTCCTTC -3’	Forward and reverse primers for methylation analysis of the cg06256735 locus using restriction endonuclease (Esp3I)
5’- GTAGGTTAAGAAAGAGGTAGAGA -3’<br>5’- CCTTCATCCTTCTTTCTCACTT -3’	Forward and reverse primers for template enrichment with the bisulfite-converted target DNA fragment before pyrosequencing (cg06256735 locus)
5’- TTGTTAGTGTTGAAGAG -3’	Sequencing primer for pyrosequencing (cg06256735 locus)
5’- GAGTTTTTTGGTTGTTGAA -3’<br>5’- CCAAATCTAAAATCCTTTC -3’	Forward and reverse primers for template enrichment with the bisulfite-converted target DNA fragment before pyrosequencing (cg15815843 locus)
5’- GTTTTTATTTTGGTTGG -3’	Sequencing primer for pyrosequencing (cg15815843 locus)
5’- GCTCTCCCTCATCTCATTGTT -3’<br>5’- CTCTTTCCACCGAGTCCTTT -3’<br>5’- HEX-CAGCGGAGGCCAAATCTGA-BHQ2 -3’	Forward and reverse primers, and probe for hydroxymethylation analysis of the cg15815843 locus using restriction endonuclease (AbaSI)
5’- ctctctatgggcagtcggtgattTAGTGAAGTTGGTAGATTGAGTTAGG -3’	Forward and reverse primers used for libraries construction before sequencing

5'- ctgcgtgtctccgactcagACTTACCACCTATAAACAACCCTTAC -3'	(5hmC-to-U conversion check). The sequences designated with uppercase letters correspond to bisulfite-converted synthetic 5hmC control DNA (cat. No. E3317S, New England Biolabs, USA), and adjacent to them sequences designated with lowercase letters correspond to the adapter sequences containing i5 and i7 index sequences (Illumina).
5'- CAGTGAAGTTGGCAGACTGAGCCAGGTCCCA CAGATGCAGTGAC**C**GGAGTCATTGCCAAACTCTGCA GGAGAGCAAGGGCTGTCTATAGGTGGCAAGTCA -3'	Synthetic 100 bp double stranded control DNA substrate – fragment containing the modification of the internal **C**= 5hmC (cat. No. E3317S, New England Biolabs, USA).

Table S9. Summary statistics

	Sample sets	N	Min	Max	Mean	SD	Median	1 quartile	3 quartile	Normality*
Paired comparisons	Esp3I_whole sample_NV [ᵻ]	30	0.57	1.00	0.86	0.12	0.87	0.76	1.00	Non-normal
	Esp3I_whole sample_VV [ᵻ]	30	0.25	1.00	0.74	0.19	0.77	0.61	0.90	Normal
	Esp3I_C2_NV [ᵻ]	19	0.68	1.00	0.90	0.12	0.99	0.77	1.00	Non-normal
	Esp3I_C2_VV [ᵻ]	19	0.45	1.00	0.78	0.16	0.78	0.68	0.91	Normal
	Esp3I_C3,4_NV [ᵻ]	11	0.57	0.90	0.78	0.10	0.80	0.72	0.87	Normal
	Esp3I_C3,4_VV [ᵻ]	11	0.25	0.97	0.68	0.24	0.71	0.48	0.89	Normal
	AbaSI_whole_NV	17	0.21	1.22	0.77	0.37	0.98	0.35	1.05	Non-normal
	AbaSI_whole_VV	17	0.40	1.51	0.98	0.24	1.02	0.93	1.07	Non-normal
	AbaSI_C2_NV	14	0.21	1.22	0.76	0.38	0.98	0.34	1.04	Non-normal
	AbaSI_C2_VV	14	0.40	1.21	0.94	0.21	1.01	0.85	1.06	Non-normal
	AbaSI_age<50_NV	11	0.21	1.22	0.74	0.38	0.97	0.34	1.04	Non-normal
	AbaSI_age<50_VV	11	0.66	1.51	1.02	0.23	1.03	0.90	1.09	Normal
	AbaSI_women_NV	10	0.21	1.16	0.64	0.37	0.49	0.33	1.02	Normal
	AbaSI_women_VV	10	0.40	1.51	0.94	0.30	1.02	0.69	1.09	Normal
	AbaSI_BMI_C2_NV	8	0.21	1.22	0.61	0.43	0.38	0.28	1.12	Non-normal
	AbaSI_BMI_C2_VV	8	0.40	1.21	0.92	0.27	1.01	0.72	1.10	Normal
	AbaSI_manifistation<50_NV	14	0.21	1.22	0.76	0.39	0.99	0.33	1.06	Non-normal
	AbaSI_manifistation<50_VV	14	0.66	1.51	1.02	0.20	1.03	0.97	1.09	Non-normal
	pyrseq_mC_cg06256735_NV	21	50.97	72.09	61.72	6.20	61.54	57.07	66.92	Normal
	pyrseq_mC_cg06256735_VV	21	33.36	54.04	43.06	6.50	41.08	37.99	47.96	Normal
	pyrseq_mC_cg15815843_NV	21	38.56	67.62	57.89	8.02	61.01	53.66	63.22	Non-normal
	pyrseq_mC_cg15815843_VV	21	26.74	47.95	38.56	6.16	40.65	32.84	43.19	Normal
	pyrseq_hmC_cg06256735_NV [ᵻ]	9	0.01	0.05	0.02	0.02	0.02	0.01	0.04	Normal
	pyrseq_hmC_cg06256735_VV [ᵻ]	9	0.01	0.13	0.07	0.04	0.07	0.04	0.10	Normal
	pyrseq_hmC_cg15815843_NV [ᵻ]	9	0.001	0.04	0.03	0.01	0.03	0.02	0.04	Normal
	pyrseq_hmC_cg15815843_VV [ᵻ]	9	0.02	0.14	0.07	0.04	0.06	0.04	0.10	Normal
	NV_I [ᵻ]	7	0.66	1.00	0.85	0.13	0.87	0.69	0.95	Normal
	VV_I [ᵻ]	7	0.49	0.88	0.66	0.16	0.72	0.50	0.80	Normal
	NV_M [ᵻ]	6	0.62	1.00	0.87	0.16	0.94	0.72	1.00	Normal
	VV_M [ᵻ]	6	0.58	1.00	0.82	0.17	0.87	0.64	0.96	Normal
	NV_A [ᵻ]	5	0.69	1.00	0.85	0.13	0.88	0.72	0.97	Normal
	VV_A [ᵻ]	5	0.72	1.00	0.90	0.11	0.94	0.81	0.97	Normal
Unpaired comparisons	Esp3I_C2_NV [ᵻ]	19	0.68	1.00	0.90	0.12	0.99	0.77	1.00	Non-normal
	Esp3I_C3,4_NV [ᵻ]	11	0.57	0.90	0.78	0.10	0.80	0.72	0.87	Normal
	NV_I [ᵻ]	7	0.66	1.00	0.85	0.13	0.87	0.69	0.95	Normal
	NV_M [ᵻ]	6	0.62	1.00	0.87	0.16	0.94	0.72	1.00	Normal
	NV_A [ᵻ]	6	0.69	1.00	0.85	0.12	0.86	0.74	0.96	Normal
	VV_I [ᵻ]	7	0.49	0.88	0.66	0.16	0.72	0.50	0.80	Normal
	VV_M [ᵻ]	6	0.58	1.00	0.82	0.17	0.87	0.64	0.96	Normal
	VV_A [ᵻ]	6	0.72	1.00	0.88	0.11	0.91	0.76	0.96	Normal

N - sample size; SD – standard deviation; NV – non-varicose vein; VV – varicose vein; C2/C3,4 – clinical status according to CEAP classification; BMI – body mass index; mC – methylcytosine; hmC – hydroxymethylcytosine; I – *t. intima*, M – *t. media*, A – *t. adventitia*.

* Normality was determined by the results of 3 tests: Shapiro-Wilk, Kolmogorov-Smirnov, and Lilliefors normality tests.

[ᵻ] Values are before conversion to percentage.

Table S10. Multiple linear regression analysis.

Method of hydroxymethyl-sensitive restriction, AbaSI (cg15815843)											
Regression model	Variable name	r	Beta	Std.Err. of Beta	B	Std.Err. of B	p (Beta)	R	R^2	p (R)	Durbin-Watson d
5hmC_NV vs. all parameters[†] (N=17)	Age	0.031						**0.514**	0.264	0.117	**2.556**
	Gender	-0.426	**-0.338**	0.240	-0.246	0.175	0.181				
	Height	0.279									
	BMI	-0.400	**-0.301**	0.240	-0.021	0.017	0.230				
	VVD manifestation	-0.020									
	CEAP class	-0.066									
	VVD duration	0.060									
5hmC_VV vs. all parameters (N=17)	Age	-0.292	**-0.456**	0.211	-0.009	0.004	0.050	**0.713**	0.509	**0.023**	1.275
	Gender	-0.208									
	Height	0.275									
	BMI	-0.476	**-0.348**	0.200	-0.016	0.009	0.105				
	VVD manifestation	-0.319									
	CEAP class	-0.410	**-0.512**	0.215	-0.307	0.129	**0.033**				
	VVD duration	0.082									
5hmC_VV:NV[‡] vs. all parameters (N=17)	Age	-0.177						**0.265**	0.070	0.304	**2.465**
	Gender	0.265	**0.265**	0.249	0.653	0.613	0.304				
	Height	-0.067									
	BMI	0.134									
	VVD manifestation	-0.156									
	CEAP class	-0.137									
	VVD duration	0.001									
5hmC_Age_whole (N=17)	~5hmC_NV	0.031						**0.292**	0.085	0.256	**1.947**
	~5hmC_VV	-0.292	**-0.292**	0.247	-14.847	12.577	0.256				
	5hmC ratio_VV:NV	-0.177									
5hmC_Age<50 y.o (N=11)	~5hmC_NV	-0.375	**-0.375**	0.309	-6.043	4.973	0.255	**0.375**	0.141	0.255	**1.866**
	~5hmC_VV	-0.305									
	5hmC ratio_VV:NV	0.209									
5hmC_BMI_whole (N=17)	~5hmC_NV	-0.400	**-0.281**	0.235	-3.951	3.303	0.251	**0.540**	0.292	0.084	1.306
	~5hmC_VV	-0.476	**-0.393**	0.235	-8.630	5.187	0.118				
	5hmC ratio_VV:NV	0.134									
5hmC_BMI_BMI>25, C2 (N=8)	~5hmC_NV	-0.220						**0.662**	0.439	0.074	**1.869**
	~5hmC_VV	-0.662	**-0.662**	0.306	-13.271	6.128	0.074				
	5hmC ratio_VV:NV	-0.214									
5hmC_VVD manifestation_whole (N=17)	~5hmC_NV	-0.020						**0.319**	0.102	0.212	0.353
	~5hmC_VV	-0.319	**-0.319**	0.245	-18.563	14.234	0.212				
	5hmC ratio_VV:NV	-0.156									
5hmC_VVD manifestation<50 y.o. (N=14)	~5hmC_NV	-0.123	No variables in the regression equation					0	0		
	~5hmC_VV	-0.090									
	5hmC ratio_VV:NV	0.111									
5hmC_Gender (N=17)	~5hmC_NV	-0.426	**-0.426**	0.234	-0.584	0.320	0.088	**0.426**	0.181	0.088	**1.756**
	~5hmC_VV	-0.208									
	5hmC ratio_VV:NV	0.265									
5hmC_CEAP (N=17)	~5hmC_NV	0.066						**0.410**	0.168	0.102	**1.893**
	~5hmC_VV	0.410	**-0.410**	0.235	-0.684	0.393	0.102				
	5hmC ratio_VV:NV	0.137									

5hmC_Height (N=17)	~5hmC_NV	0.279	**0.671**	0.425	13.590	8.598	0.136	**0.394**	0.160	0.310	**2.275**
	~5hmC_VV	0.274									
	5hmC ratio_VV:NV	-0.067	**0.480**	0.425	2.881	2.546	0.277				
5hmC_VVD duration (N=17)	~5hmC_NV	0.060	No variables in the regression equation					0	0		
	~5hmC_VV	0.082									
	5hmC ratio_VV:NV	0.001									
**Ox/BS-pyrosequencing (cg06256735)**											
Regression model	Variable name	r	Beta	Std.Err. of Beta	B	Std.Err. of B	p (Beta)	R	R^2	p (R)	Durbin-Watson d
5hmC_NV vs. all parameters (N=9)	Age	0.785	**0.785**	0.234	0.110	0.033	**0.012**	**0.785**	0.617	**0.012**	**1.919**
	Gender	-0.377									
	Height	0.123									
	BMI	0.582									
	VVD manifestation	0.438									
	CEAP class	0.411									
	VVD duration	0.575									
5hmC_VV vs. all parameters (N=9)	Age	-0.468						**0.479**	0.229	0.192	1.280
	Gender	0.067									
	Height	0.103									
	BMI	-0.322									
	VVD manifestation	-0.076									
	CEAP class	-0.049									
	VVD duration	-0.479	**-0.479**	0.332	-0.170	0.118	0.192				
5hmC_VV:NV vs. all parameters (N=9)	Age	-0.544						**0.744**	0.553	0.089	**1.612**
	Gender	0.330	**0.492**	0.281	4.822	2.755	0.131				
	Height	-0.088									
	BMI	-0.570	**-0.686**	0.281	-1.120	0.459	0.050				
	VVD manifestation	-0.245									
	CEAP class	-0.245									
	VVD duration	-0.442									
5hmC_Age (N=9)	% 5hmC_NV	0.785	**1.061**	0.272	7.570	1.942	**0.011**	**0.909**	0.827	**0.024**	**2.735**
	% 5hmC_VV	-0.468	**-0.845**	0.351	-2.710	1.127	0.061				
	5hmC ratio_VV:NV	-0.544	**0.738**	0.427	1.873	1.083	0.144				
5hmC_BMI (N=9)	% 5hmC_NV	0.582	**0.582**	0.307	1.001	0.529	0.100	**0.582**	0.338	0.100	**2.029**
	% 5hmC_VV	-0.322									
	5hmC ratio_VV:NV	-0.570									
5hmC_Gender (N=9)	% 5hmC_NV	-0.377	**-0.377**	0.350	-0.108	0.100	0.317	**0.377**	0.142	0.317	1.539
	% 5hmC_VV	0.067									
	5hmC ratio_VV:NV	0.330									
5hmC_VVD manifestation (N=9)	% 5hmC_NV	0.438	**0.438**	0.340	2.007	1.557	0.238	**0.438**	0.192	0.238	**2.945**
	% 5hmC_VV	-0.076									
	5hmC ratio_VV:NV	-0.245									
5hmC_CEAP (N=9)	% 5hmC_NV	0.411	**-0.411**	0.345	-0.104	0.087	0.272	**0.411**	0.169	0.272	**2.380**
	% 5hmC_VV	-0.049									
	5hmC ratio_VV:NV	-0.245									
5hmC_VVD duration (N=9)	% 5hmC_NV	0.575	**0.875**	0.415	5.466	2.591	0.089	**0.774**	0.599	0.175	**2.953**
	% 5hmC_VV	-0.479	**-0.947**	0.535	-2.660	1.504	0.137				
	5hmC ratio_VV:NV	-0.442	**0.810**	0.650	1.801	1.445	0.268				
5hmC_Height (N=9)	% 5hmC_NV	0.123	No variables in the regression equation					0	0		
	% 5hmC_VV	0.103									
	5hmC ratio_VV:NV	-0.088									

Ox/BS-pyrosequencing (cg15815843)											
Regression model	Variable name	r	Beta	Std.Err. of Beta	B	Std.Err. of B	p (Beta)	R	R^2	p (R)	Durbin-Watson d
5hmC_NV vs. all parameters (N=9)	Age	0.393						**0.715**	0.511	0.117	**1.909**
	Gender	-0.653	**-0.636**	0.286	-1.517	0.682	0.068				
	Height	0.618									
	BMI	0.173									
	VVD manifestation	0.440									
	CEAP class	0.328	**-0.290**	0.286	-0.828	0.815	0.349				
	VVD duration	-0.043									
5hmC_VV vs. all parameters (N=9)	Age	-0.343	**-0.521**	0.243	-0.144	0.067	0.099	**0.917**	0.841	0.068	**1.669**
	Gender	0.034	**0.548**	0.353	4.116	2.651	0.195				
	Height	0.369	**0.902**	0.324	0.354	0.127	**0.0496**				
	BMI	-0.079									
	VVD manifestation	-0.176									
	CEAP class	0.453	**-0.685**	0.214	-6.154	1.924	**0.033**				
	VVD duration	-0.211									
5hmC_VV:NV vs. all parameters (N=9)	Age	-0.284						**0.536**	0.287	0.137	**1.741**
	Gender	0.389									
	Height	-0.536	**-0.536**	0.319	-0.393	0.234	0.137				
	BMI	-0.219									
	VVD manifestation	-0.355									
	CEAP class	-0.109									
	VVD duration	0.076									
5hmC_Age (N=9)	% 5hmC_NV	0.393	**0.656**	0.328	7.495	3.746	0.092	**0.686**	0.471	0.148	1.012
	% 5hmC_VV	-0.343	**-0.621**	0.328	-2.253	1.190	0.107				
	5hmC ratio_VV:NV	-0.284									
5hmC_BMI (N=9)	% 5hmC_NV	0.173	No variables in the regression equation					0	0		
	% 5hmC_VV	-0.079									
	5hmC ratio_VV:NV	-0.219									
5hmC_Gender (N=9)	% 5hmC_NV	-0.653	**-0.814**	0.304	-0.341	0.127	**0.037**	**0.738**	0.545	0.094	**2.205**
	% 5hmC_VV	0.034	**0.380**	0.304	0.051	0.040	0.258				
	5hmC ratio_VV:NV	0.389									
5hmC_VVD manifestation (N=9)	% 5hmC_NV	0.440	**0.627**	0.362	6.973	4.032	0.134	**0.594**	0.353	0.270	**2.796**
	% 5hmC_VV	-0.176	**-0.442**	0.362	-1.560	1.280	0.269				
	5hmC ratio_VV:NV	-0.355									
5hmC_CEAP class (N=9)	% 5hmC_NV	0.328						**0.453**	0.206	0.220	0.986
	% 5hmC_VV	0.453	**-0.453**	0.337	-0.051	0.038	0.220				
	5hmC ratio_VV:NV	-0.109									
5hmC_VVD duration (N=9)	% 5hmC_NV	-0.043	No variables in the regression equation					0	0		
	% 5hmC_VV	-0.211									
	5hmC ratio_VV:NV	0.076									
5hmC_Height (N=9)	% 5hmC_NV	0.618	**0.618**	0.297	4.961	2.387	0.076	**0.618**	0.382	0.076	**2.270**
	% 5hmC_VV	0.369									
	5hmC ratio_VV:NV	-0.536									

Method of methyl-sensitive restriction, ESP3I (cg06256735)											
Regression model	Variable name	r	Beta	Std.Err. of Beta	B	Std.Err. of B	p (Beta)	R	R^2	p (R)	Durbin-Watson d
5mC_NV vs. all parameters (N=28)	Age	-0.106						**0.489**	0.239	**0.033**	**2.165**
	Gender	0.130	0.219	0.177	5.822	4.714	0.228				
	Height	0.067									
	BMI	-0.317									

	VVD manifestation	-0.150									
	CEAP class	-0.439	**-0.480**	0.177	-5.894	2.180	**0.012**				
	VVD duration	0.015									
5mC_VV vs. all parameters (N=28)	Age	-0.051						**0.287**	0.082	0.343	1.525
	Gender	0.136									
	Height	0.132									
	BMI	-0.193									
	VVD manifestation	-0.175									
	CEAP class	-0.205	**-0.286**	0.207	-5.548	4.008	0.179				
	VVD duration	0.109	0.216	0.207	0.400	0.382	0.305				
5mC_NV:VV vs. all parameters (N=28)	Age	-0.091	No variables in the regression equation					0	0		
	Gender	0.007									
	Height	-0.101									
	BMI	0.058									
	VVD manifestation	-0.034									
	CEAP class	0.137									
	VVD duration	-0.079									
5mC_Age (N=30)	% 5mC_NV	-0.136	No variables in the regression equation					0	0		
	% 5mC_VV	-0.066									
	5mC ratio_NV:VV	-0.089									
5mC_BMI (N=28)	% 5mC_NV	-0.317	**-0.317**	0.186	-0.132	0.077	0.101	**0.317**	0.100	0.101	**2.062**
	% 5mC_VV	-0.193									
	5mC ratio_NV:VV	0.058									
5mC_Gender (N=30)	% 5mC_NV	-0.137	No variables in the regression equation					0	0		
	% 5mC_VV	-0.141									
	5mC ratio_NV:VV	0.003									
5mC_VVD manifestation (N=30)	% 5mC_NV	-0.173	No variables in the regression equation					0	0		
	% 5mC_VV	-0.185									
	5mC ratio_NV:VV	-0.038									
5mC_CEAP (N=30)	% 5mC_NV	-0.431	**-0.695**	0.228	-0.056	0.018	**0.005**	**0.544**	0.296	**0.026**	**2.351**
	% 5mC_VV	-0.209	**0.500**	0.366	0.026	0.019	0.183				
	5mC ratio_NV:VV	0.146	**0.692**	0.365	1.317	0.694	0.069				
5mC_Height (N=28)	% 5mC_NV	0.067	No variables in the regression equation					0	0		
	% 5mC_VV	0.132									
	5mC ratio_NV:VV	-0.101									
5mC_VVD duration (N=30)	% 5mC_NV	0.001	No variables in the regression equation					0	0		
	% 5mC_VV	0.100									
	5mC ratio_NV:VV	-0.074									

BS-pyrosequencing (cg06256735)

Regression model	Variable name	r	Beta	Std.Err. of Beta	B	Std.Err. of B	p (Beta)	R	R^2	p (R)	Durbin-Watson d
5mC_NV vs. all parameters (N=21)	Age	-0.161						**0.762**	0.580	**0.002**	**1.959**
	Gender	0.587	**0.711**	0.174	9.528	2.332	**0.001**				
	Height	0.404									
	BMI	-0.299									
	VVD manifestation	-0.184	**-0.394**	0.179	-0.206	0.094	**0.042**				
	CEAP class	-0.402	-0.224	0.167	-1.593	1.187	0.197				
	VVD duration	0.026									
5mC_VV vs. all parameters (N=21)	Age	-0.385	**-0.413**	0.214	-0.224	0.116	0.072	**0.613**	0.376	0.092	**2.106**
	Gender	0.278	**0.632**	0.310	8.881	4.359	0.058				
	Height	0.097	**-0.398**	0.307	-0.313	0.241	0.213				

	BMI	-0.351	-0.225	0.212	-0.278	0.263	0.306				
	VVD manifestation	-0.091									
	CEAP class	-0.108									
	VVD duration	-0.356									
5mC_NV:VV<br>vs.<br>all parameters<br>(N=21)	Age	0.304						**0.484**	0.234	0.091	**2.132**
	Gender	0.086									
	Height	0.135	0.249	0.212	0.007	0.006	0.256				
	BMI	0.179									
	VVD manifestation	-0.044									
	CEAP class	-0.168									
	VVD duration	0.419	**0.478**	0.212	0.011	0.005	**0.037**				
5mC_Age<br>(N=21)	% 5mC_NV	-0.161						**0.385**	0.148	**0.085**	**1.832**
	% 5mC_VV	-0.385	**-0.385**	0.212	-0.709	0.390	0.085				
	5mC ratio_NV:VV	0.304									
5mC_BMI<br>(N=21)	% 5mC_NV	-0.299						**0.351**	0.123	**0.118**	**2.442**
	% 5mC_VV	-0.351	**-0.351**	0.215	-0.284	0.174	0.118				
	5mC ratio_NV:VV	0.179									
5mC_Gender<br>(N=21)	% 5mC_NV	0.587	**0.587**	0.186	0.044	0.014	**0.005**	**0.587**	0.345	**0.005**	1.475
	% 5mC_VV	0.278									
	5mC ratio_NV:VV	0.086									
5mC_VVD manifestation<br>(N=21)	% 5mC_NV	-0.184	No variables in the regression equation					0	0		
	% 5mC_VV	-0.091									
	5mC ratio_NV:VV	-0.044									
5mC_CEAP<br>(N=21)	% 5mC_NV	-0.402	**-0.402**	0.210	-0.057	0.030	0.071	**0.402**	0.162	0.071	**2.186**
	% 5mC_VV	-0.108									
	5mC ratio_NV:VV	-0.168									
5mC_Height<br>(N=21)	% 5mC_NV	0.404	**0.404**	0.210	0.539	0.280	0.069	**0.404**	0.163	0.069	**2.525**
	% 5mC_VV	0.097									
	5mC ratio_NV:VV	0.135									
5mC_VVD duration<br>(N=21)	% 5mC_NV	0.026						**0.419**	0.175	0.059	**2.124**
	% 5mC_VV	-0.356									
	5mC ratio_NV:VV	0.419	**0.419**	0.208	18.665	9.281	0.059				
**BS-pyrosequencing (cg15815843)**											
Regression model	Variable name	r	Beta	Std.Err. of Beta	B	Std.Err. of B	p (Beta)	R	R^2	p (R)	Durbin-Watson d
5mC_NV<br>vs.<br>all parameters<br>(N=21)	Age	-0.250						**0.762**	0.580	**0.005**	**2.063**
	Gender	-0.435	**-0.492**	0.185	-8.531	3.198	**0.017**				
	Height	0.292									
	BMI	-0.440	**-0.313**	0.170	-0.478	0.260	0.084				
	VVD manifestation	-0.289	**-0.334**	0.193	-0.226	0.130	0.102				
	CEAP class	-0.439	**-0.298**	0.173	-5.481	3.184	0.104				
	VVD duration	0.043									
5mC_VV<br>vs.<br>all parameters<br>(N=21)	Age	-0.317	**-0.297**	0.215	-0.152	0.111	0.186	**0.571**	0.326	0.076	1.490
	Gender	-0.325	**-0.348**	0.207	-4.625	2.748	0.111				
	Height	0.320									
	BMI	-0.417	**-0.286**	0.213	-0.334	0.249	0.197				
	VVD manifestation	-0.104									
	CEAP class	-0.006									
	VVD duration	-0.257									
5mC_NV:VV<br>vs.	Age	0.045						**0.590**	0.349	**0.021**	**1.852**
	Gender	-0.029									

all parameters (N=21)	Height	-0.081									
	BMI	0.032									
	VVD manifestation	-0.240									
	CEAP class	-0.430	**-0.487**	0.192	-0.252	0.100	**0.021**				
	VVD duration	0.341	**0.409**	0.192	0.009	0.004	**0.048**				
	% 5mC_NV	-0.250									
5mC_Age (N=21)	% 5mC_VV	-0.317	**-0.317**	0.218	-0.616	0.423	0.162	**0.317**	0.100	0.162	**1.794**
	5mC ratio_NV:VV	0.045									
	% 5mC_NV	-0.440	**-0.304**	0.243	-0.199	0.159	0.226				
5mC_BMI (N=21)	% 5mC_VV	-0.417	**-0.254**	0.243	-0.217	0.207	0.309	**0.490**	0.240	0.085	**1.641**
	5mC ratio_NV:VV	0.032									
	% 5mC_NV	-0.435	**-0.435**	0.207	-0.025	0.012	**0.049**				
5mC_Gender (N=21)	% 5mC_VV	-0.325						**0.435**	0.190	**0.049**	1.318
	5mC ratio_NV:VV	-0.029									
	% 5mC_NV	-0.289	**-0.289**	0.220	-0.428	0.325	0.204				
5mC_VVD manifestation (N=21)	% 5mC_VV	-0.104						**0.289**	0.084	0.204	**2.139**
	5mC ratio_NV:VV	-0.240									
	% 5mC_NV	-0.439	**-0.324**	0.217	-0.018	0.012	0.152				
5mC_CEAP (N=21)	% 5mC_VV	-0.006						**0.524**	0.275	0.055	**2.473**
	5mC ratio_NV:VV	-0.430	**-0.309**	0.217	-0.595	0.417	0.171				
	% 5mC_NV	0.292									
5mC_Height (N=21)	% 5mC_VV	0.320	**0.320**	0.217	0.431	0.292	0.157	**0.320**	0.103	0.157	**2.019**
	5mC ratio_NV:VV	-0.081									
	% 5mC_NV	0.043									
5mCg2_VVD duration (N=21)	% 5mC_VV	-0.257						**0.341**	0.116	0.130	**2.077**
	5mC ratio_NV:VV	0.341	**0.341**	0.216	14.957	9.454	0.130				

N – sample size; NV – non-varicose vein; VV – varicose vein; C2/C3,4 – clinical status according to CEAP classification; BMI – body mass index; 5mC – 5-methylcytosine; 5hmC – 5-hydroxymethylcytosine; VVD – varicose vein disease; Std.Err. – standard error; r – correlation coefficient; R – multiple correlation coefficient; R^2 – coefficient of determination; p – p-value.

Beta – Beta>|±0.25| and p (Beta)>0.05; **Beta** – Beta>|±0.25| and p (Beta)<0.05; **p (Beta)** – p (Beta)<0.05; **R** – R>|±0.25| and p (R)>0.05; **R** – R>|±0.25| and p (R)<0.05; **p (R)** – p (R)<0.05; **Durbin-Watson d>1.586** (autocorrelation of residuals is absent); Durbin-Watson d<1.586 (autocorrelation of residuals is present).

† all parameters (independent predictor variables) applied together: Age, BMI, Gender, VVD manifestation, CEAP class, Height, VVD duration.

‡ NV:VV or VV:NV ratio.

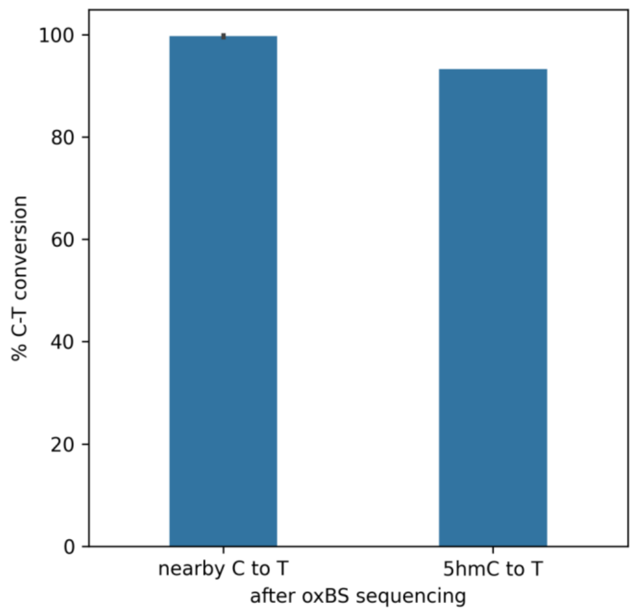

Figure S1. C-to-T conversion levels determined by means of Illumina sequencing of synthetic 5hmC-dsDNA after oxidative bisulfite treatment

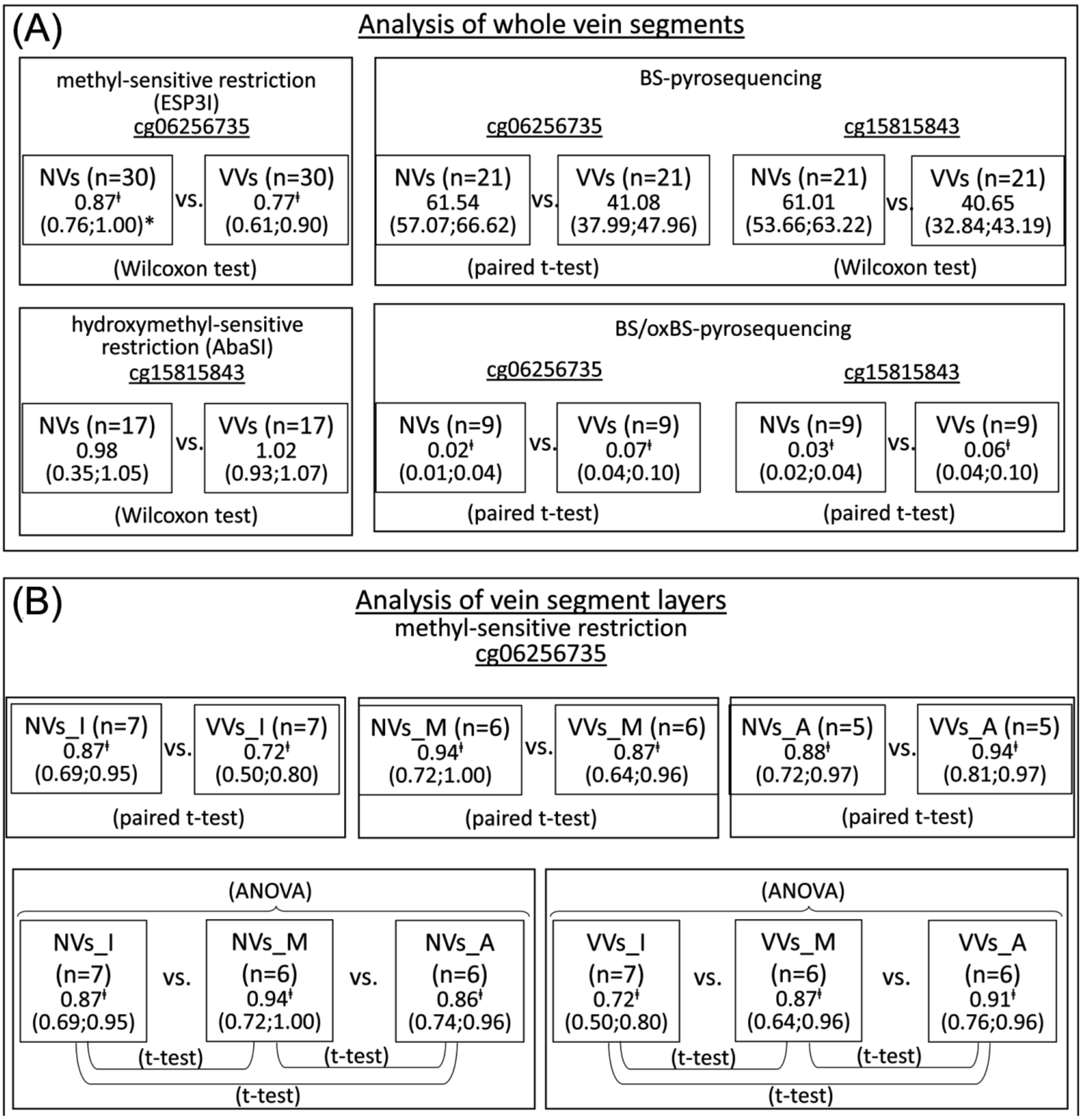

Figure S2. Statistical analysis strategy and brief summary statistics outlined. NV – non-varicose veins; VV – varicose veins; C2 and C3,4 – clinical status of patient according to the CEAP classification; 'I' – *t. intima*, 'M' – *t. media*, 'A' – *t. adventitia*, ANOVA – analysis of variance, * Wilcoxon test or paired Student's t-test. The choice of statistical test depended on the normality of a distribution. ǂ Values are before conversion to percentage.

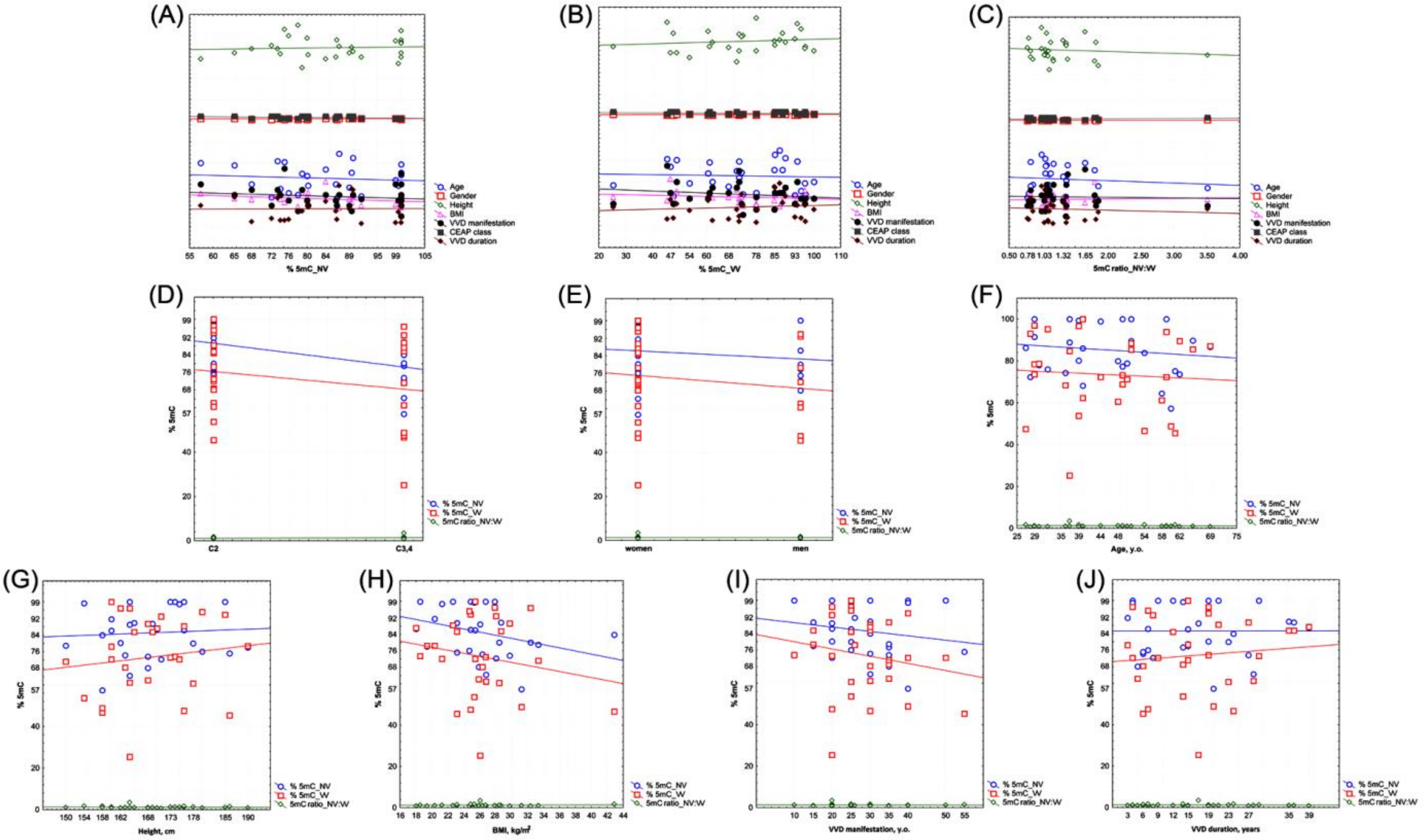

Figure S3. Correlation scatterplots complementary to Figure 3F (the result of MLR analysis for the cg06256735 locus methylation data obtained using the method of methyl-sensitive restriction, Esp3I)

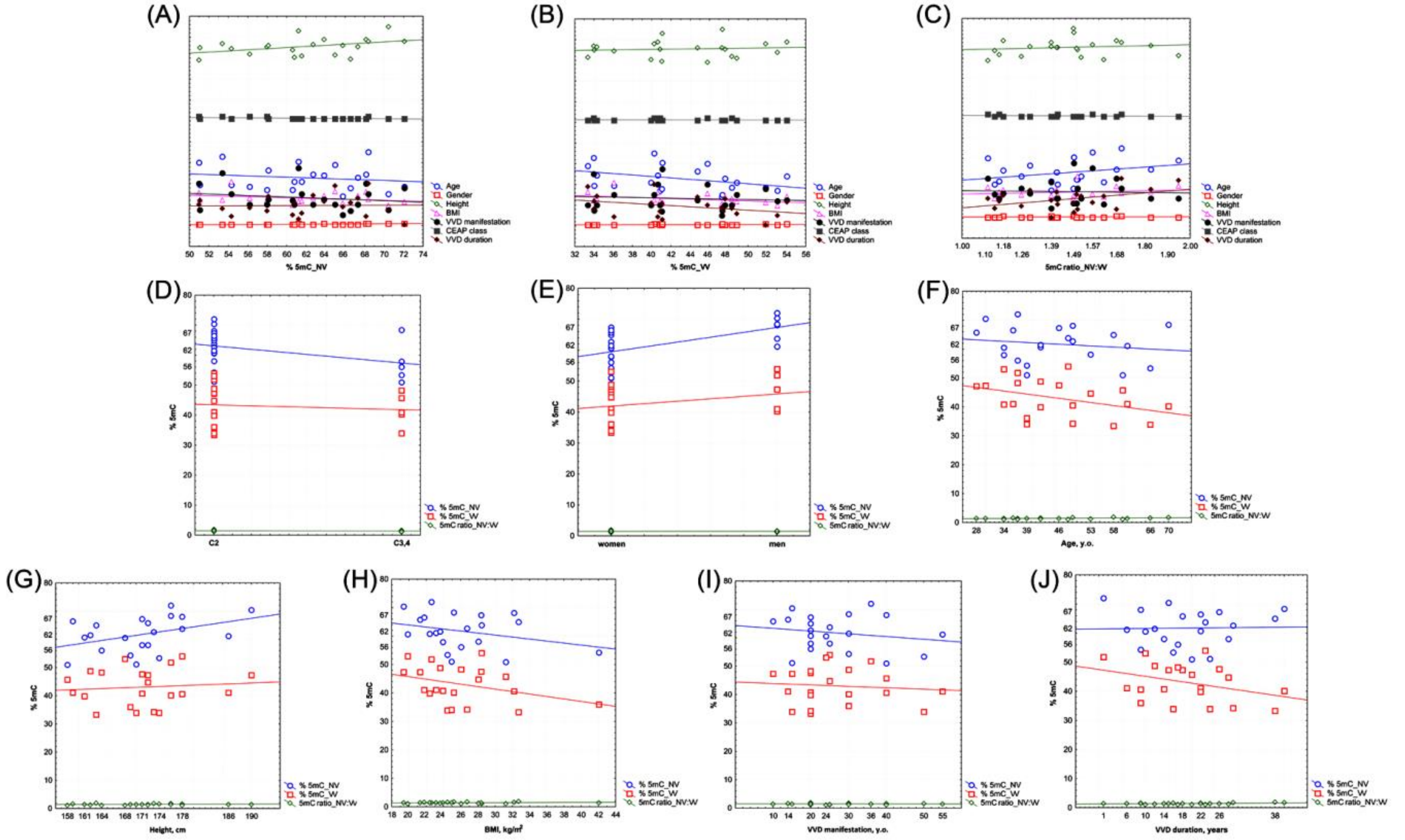

Figure S4. Correlation scatterplots complementary to Figure 3G (the result of MLR analysis for the cg06256735 locus methylation data obtained by BS-pyrosequencing)

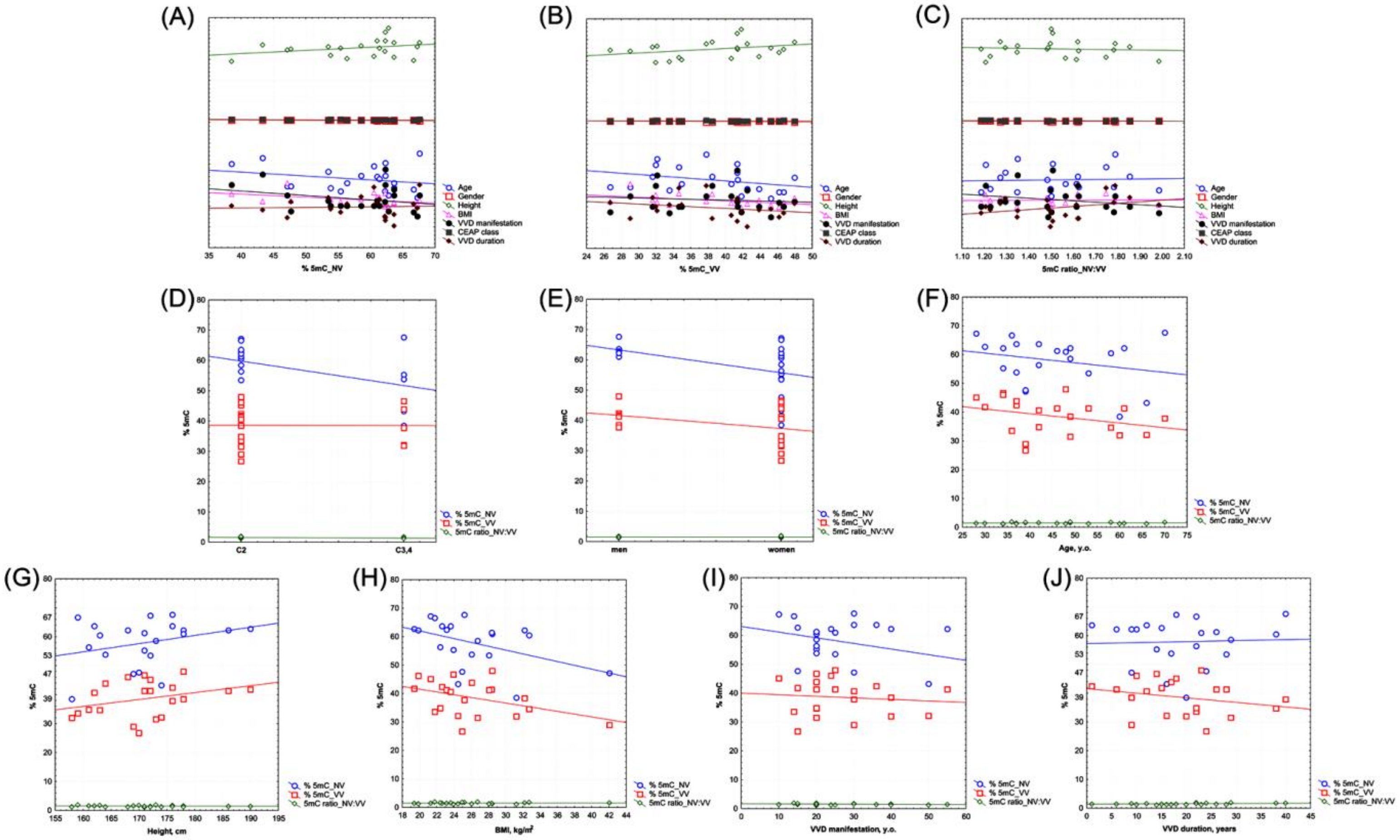

Figure S5. Correlation scatterplots complementary to Figure 3H (the result of MLR analysis for the cg15815843 locus methylation data obtained by BS-pyrosequencing)

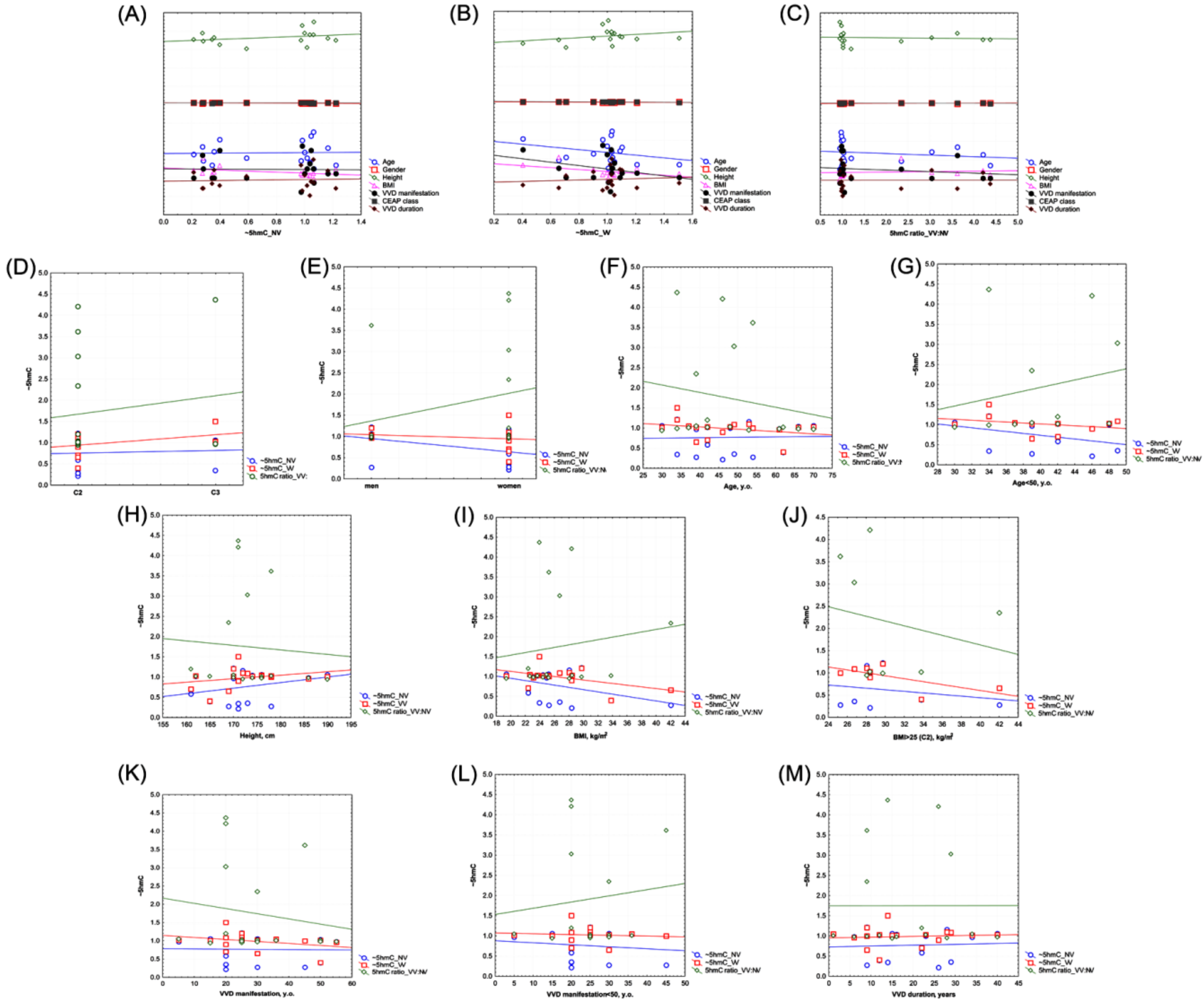

Figure S6. Correlation scatterplots complementary to Figure 4G (the result of MLR analysis for the cg15815843 locus hydroxymethylation data obtained using the method of hydroxymethyl-sensitive restriction (AbaSI)

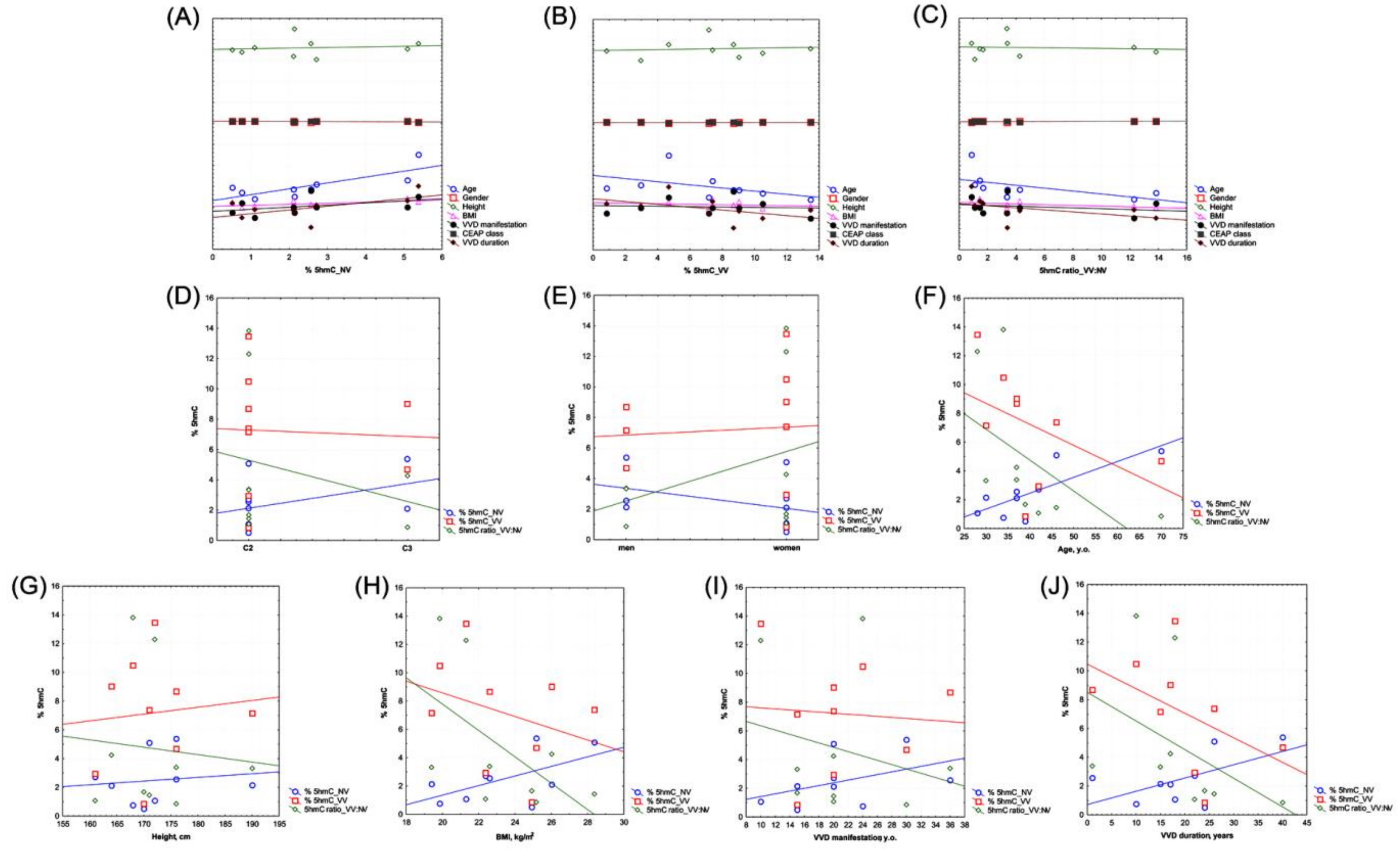

Figure S7. Correlation scatterplots complementary to Figure 5D (the result of MLR analysis for the cg06256735 locus hydroxymethylation data)

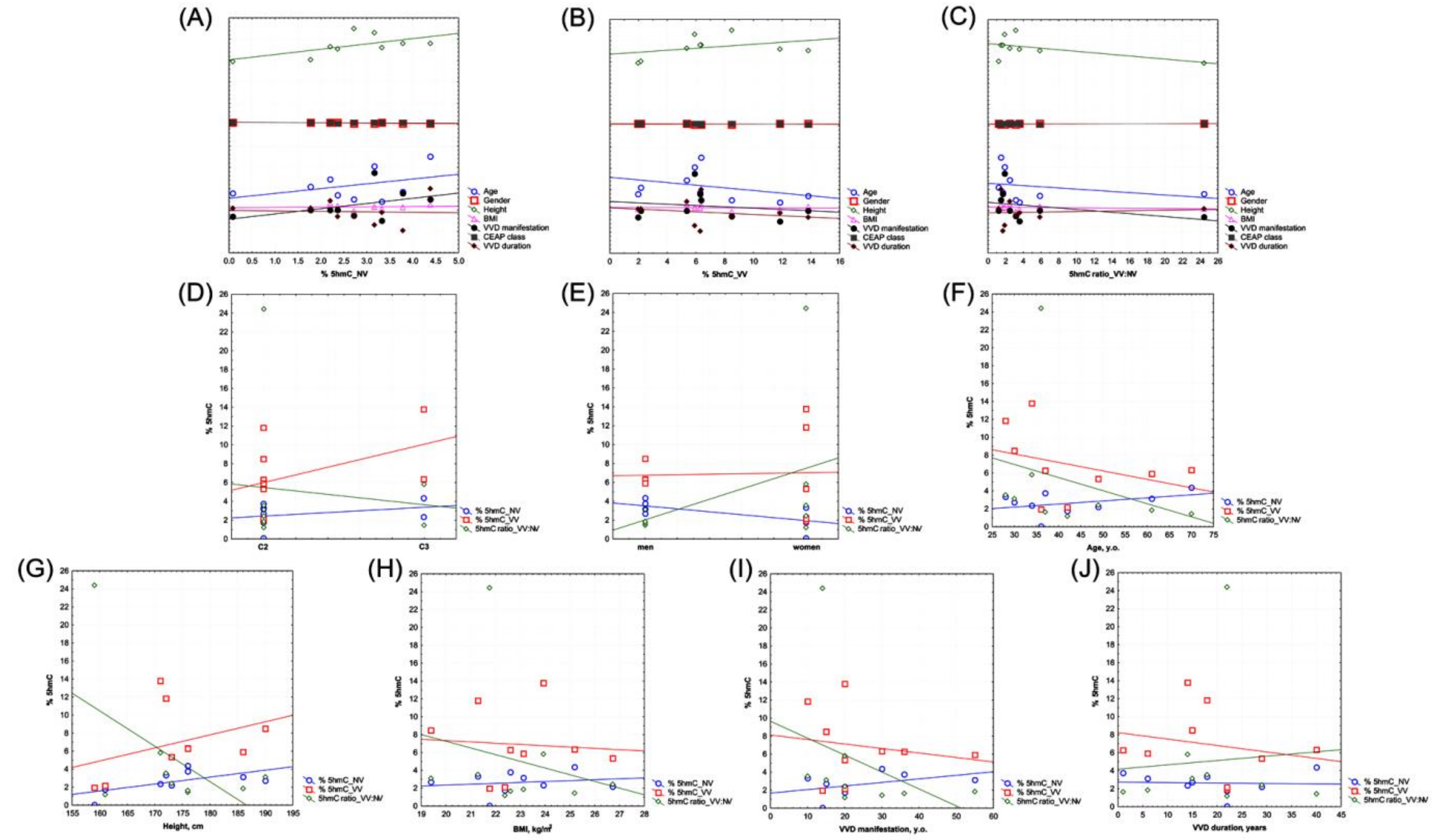

Figure S8. Correlation scatterplots complementary to Figure 5E (the result of MLR analysis for the cg15815843 locus hydroxymethylation data)
